# Supplementary figures and images for: The effect of target transpulmonary driving pressure values on mortality in ARDS patients: A retrospective study based on the MIMIC-IV database
Source: PLoS One. 2025 Jun 18;20(6):e0326060. doi: 10.1371/journal.pone.0326060 (PMC12176163; doi:10.1371/journal.pone.0326060)

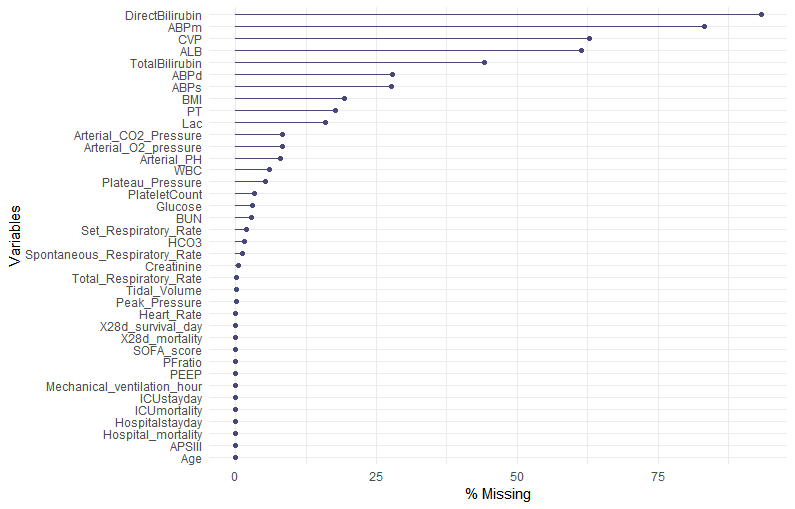

Supplement: S1 Fig — (TIF) [file pone.0326060.s001.tif]

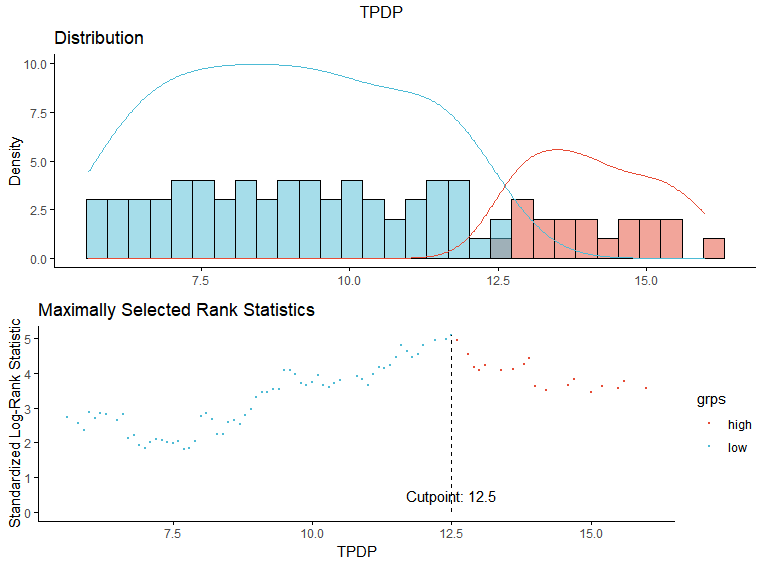

Supplement: S2 Fig — TPDP, Transpulmonary driving pressure; grps, groups. (TIF) [file pone.0326060.s002.tif]

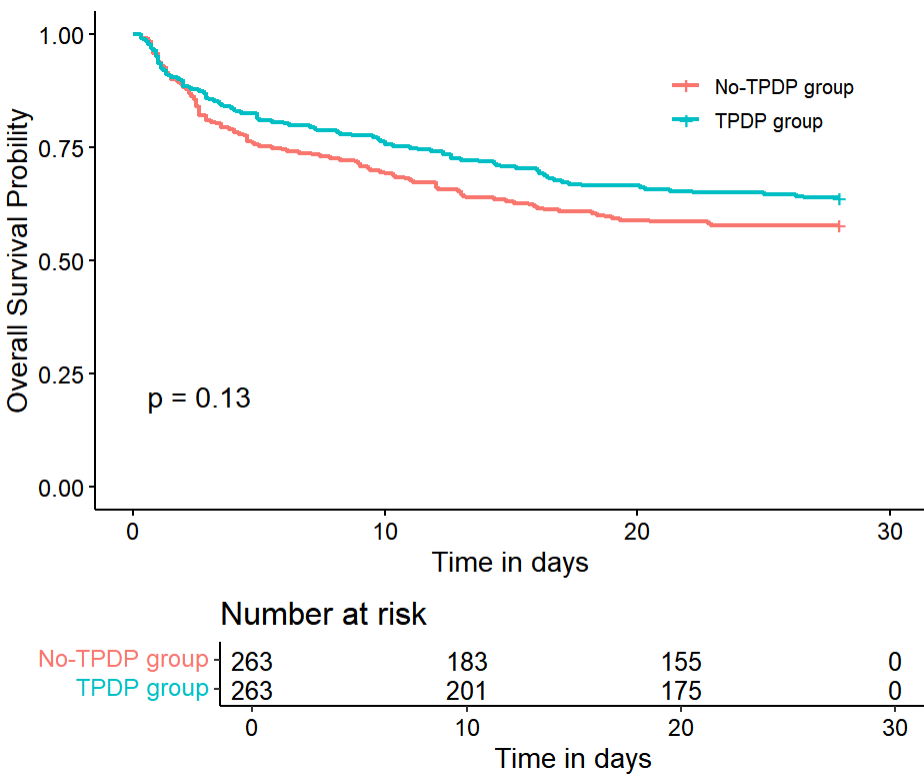

Supplement: S3 Fig — (TIF) [file pone.0326060.s003.tif]

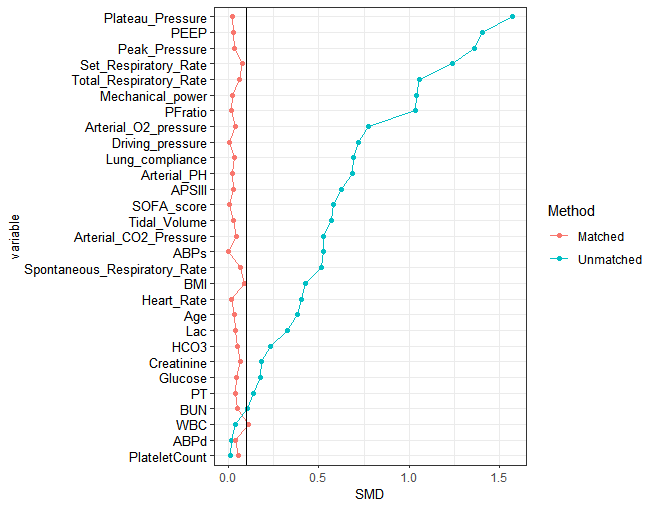

Supplement: S4 Fig — PEEP, Positive end expiratory pressure; PFratio, P/F ratio of the first day; APSIII, Acute physiology score; ABPs, Systolic arterial pressure; BMI, Body mass index; Lac, Lactate level; PT, Prothrombin time; BUN, Urea nitrogen; WBC, White blood cell; ABPd, Diastolic blood pressure. (TIF) [file pone.0326060.s004.tif]

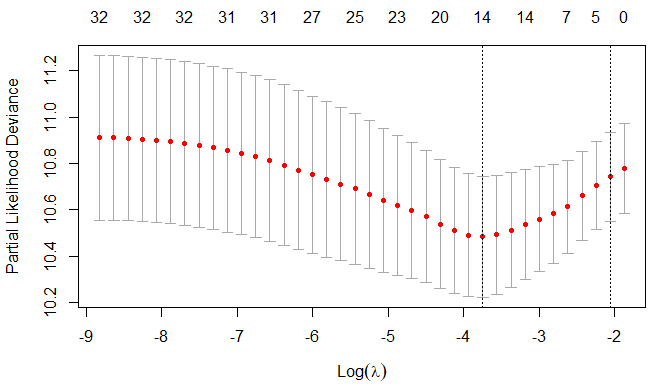

Supplement: S5 Fig — (TIF) [file pone.0326060.s005.tif]

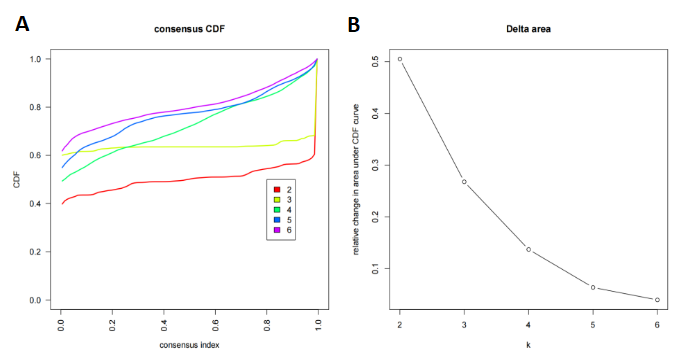

Supplement: S6 Fig — (TIF) [file pone.0326060.s006.tif]

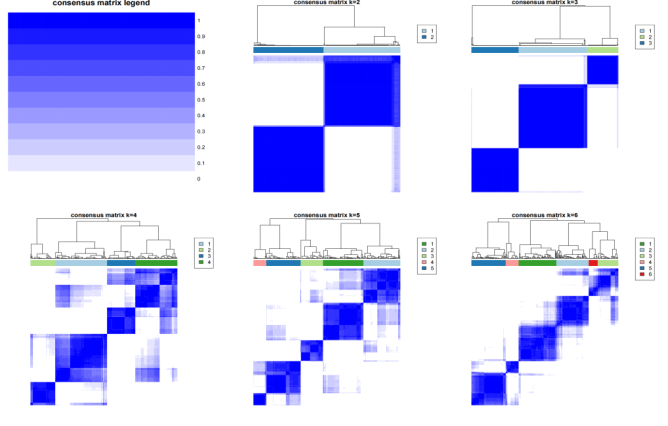

Supplement: S7 Fig — (TIF) [file pone.0326060.s007.tif]

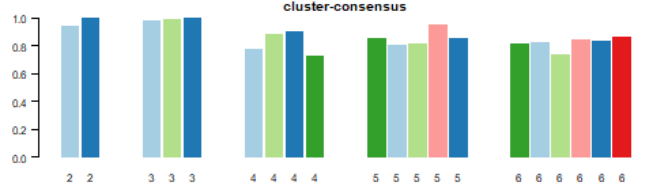

Supplement: S8 Fig — (TIF) [file pone.0326060.s008.tif]

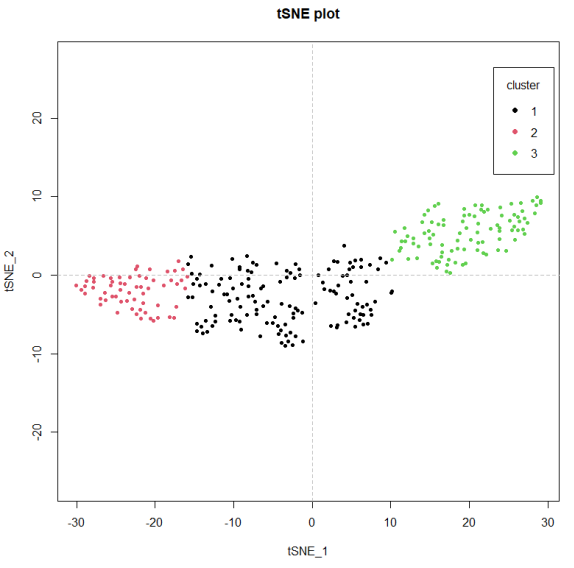

Supplement: S9 Fig — (TIF) [file pone.0326060.s009.tif]
