# Supplementary material for: The effect of target transpulmonary driving pressure values on mortality in ARDS patients: A retrospective study based on the MIMIC-IV database
Source: PLoS One. 2025 Jun 18;20(6):e0326060. doi: 10.1371/journal.pone.0326060 (PMC12176163; doi:10.1371/journal.pone.0326060)
Supplement: S1 Table — (DOCX) [file pone.0326060.s010.docx]

**eTable 1** Normality test of variables between groups.

Normality test of variables in TPDP Group and No-TPDP Group.

| Variable | Test_Scope | W_statistic | P_value | N |
| --- | --- | --- | --- | --- |
| Age | All | 0.984 | 0.000 | 4721 |
| Age | No-TPDP group | 0.983 | 0.000 | 4426 |
| Age | TPDP group | 0.984 | 0.002 | 295 |
| BMI | All | 0.899 | 0.000 | 4721 |
| BMI | No-TPDP group | 0.902 | 0.000 | 4426 |
| BMI | TPDP group | 0.907 | 0.000 | 295 |
| SOFA_score | All | 0.968 | 0.000 | 4721 |
| SOFA_score | No-TPDP group | 0.965 | 0.000 | 4426 |
| SOFA_score | TPDP group | 0.988 | 0.012 | 295 |
| APSIII | All | 0.980 | 0.000 | 4721 |
| APSIII | No-TPDP group | 0.980 | 0.000 | 4426 |
| APSIII | TPDP group | 0.990 | 0.047 | 295 |
| ABPd | All | 0.983 | 0.000 | 4721 |
| ABPd | No-TPDP group | 0.982 | 0.000 | 4426 |
| ABPd | TPDP group | 0.987 | 0.008 | 295 |
| ABPs | All | 0.962 | 0.000 | 4721 |
| ABPs | No-TPDP group | 0.962 | 0.000 | 4426 |
| ABPs | TPDP group | 0.967 | 0.000 | 295 |
| Heart_Rate | All | 0.993 | 0.000 | 4721 |
| Heart_Rate | No-TPDP group | 0.993 | 0.000 | 4426 |
| Heart_Rate | TPDP group | 0.992 | 0.088 | 295 |
| WBC | All | 0.990 | 0.000 | 4721 |
| WBC | No-TPDP group | 0.990 | 0.000 | 4426 |
| WBC | TPDP group | 0.988 | 0.019 | 295 |
| Creatinine | All | 0.633 | 0.000 | 4721 |
| Creatinine | No-TPDP group | 0.639 | 0.000 | 4426 |
| Creatinine | TPDP group | 0.564 | 0.000 | 295 |
| Glucose | All | 0.985 | 0.000 | 4721 |
| Glucose | No-TPDP group | 0.985 | 0.000 | 4426 |
| Glucose | TPDP group | 0.982 | 0.001 | 295 |
| BUN | All | 0.900 | 0.000 | 4721 |
| BUN | No-TPDP group | 0.899 | 0.000 | 4426 |
| BUN | TPDP group | 0.916 | 0.000 | 295 |
| PT | All | 0.920 | 0.000 | 4721 |
| PT | No-TPDP group | 0.918 | 0.000 | 4426 |
| PT | TPDP group | 0.949 | 0.000 | 295 |
| PlateletCount | All | 0.967 | 0.000 | 4721 |
| PlateletCount | No-TPDP group | 0.967 | 0.000 | 4426 |
| PlateletCount | TPDP group | 0.967 | 0.000 | 295 |
| Lac | All | 0.939 | 0.000 | 4721 |
| Lac | No-TPDP group | 0.940 | 0.000 | 4426 |
| Lac | TPDP group | 0.936 | 0.000 | 295 |
| Arterial_PH | All | 0.854 | 0.000 | 4721 |
| Arterial_PH | No-TPDP group | 0.850 | 0.000 | 4426 |
| Arterial_PH | TPDP group | 0.836 | 0.000 | 295 |
| Arterial_O2_pressure | All | 0.970 | 0.000 | 4721 |
| Arterial_O2_pressure | No-TPDP group | 0.971 | 0.000 | 4426 |
| Arterial_O2_pressure | TPDP group | 0.951 | 0.000 | 295 |
| Arterial_CO2_Pressure | All | 0.993 | 0.000 | 4721 |
| Arterial_CO2_Pressure | No-TPDP group | 0.993 | 0.000 | 4426 |
| Arterial_CO2_Pressure | TPDP group | 0.990 | 0.049 | 295 |
| HCO3 | All | 0.992 | 0.000 | 4721 |
| HCO3 | No-TPDP group | 0.992 | 0.000 | 4426 |
| HCO3 | TPDP group | 0.988 | 0.017 | 295 |
| PFratio | All | 0.960 | 0.000 | 4721 |
| PFratio | No-TPDP group | 0.961 | 0.000 | 4426 |
| PFratio | TPDP group | 0.899 | 0.000 | 295 |
| Total_Respiratory_Rate | All | 0.984 | 0.000 | 4721 |
| Total_Respiratory_Rate | No-TPDP group | 0.984 | 0.000 | 4426 |
| Total_Respiratory_Rate | TPDP group | 0.978 | 0.000 | 295 |
| Spontaneous_Respiratory_Rate | All | 0.702 | 0.000 | 4721 |
| Spontaneous_Respiratory_Rate | No-TPDP group | 0.715 | 0.000 | 4426 |
| Spontaneous_Respiratory_Rate | TPDP group | 0.427 | 0.000 | 295 |
| Set_Respiratory_Rate | All | 0.972 | 0.000 | 4721 |
| Set_Respiratory_Rate | No-TPDP group | 0.972 | 0.000 | 4426 |
| Set_Respiratory_Rate | TPDP group | 0.966 | 0.000 | 295 |
| Tidal_Volume | All | 1.000 | 0.671 | 4721 |
| Tidal_Volume | No-TPDP group | 1.000 | 0.746 | 4426 |
| Tidal_Volume | TPDP group | 0.996 | 0.571 | 295 |
| PEEP | All | 0.828 | 0.000 | 4721 |
| PEEP | No-TPDP group | 0.826 | 0.000 | 4426 |
| PEEP | TPDP group | 0.889 | 0.000 | 295 |
| Plateau_Pressure | All | 0.984 | 0.000 | 4721 |
| Plateau_Pressure | No-TPDP group | 0.987 | 0.000 | 4426 |
| Plateau_Pressure | TPDP group | 0.987 | 0.009 | 295 |
| Peak_Pressure | All | 0.992 | 0.000 | 4721 |
| Peak_Pressure | No-TPDP group | 0.993 | 0.000 | 4426 |
| Peak_Pressure | TPDP group | 0.991 | 0.069 | 295 |
| Lung_compliance | All | 0.722 | 0.000 | 4721 |
| Lung_compliance | No-TPDP group | 0.710 | 0.000 | 4426 |
| Lung_compliance | TPDP group | 0.881 | 0.000 | 295 |
| Driving_pressure | All | 0.983 | 0.000 | 4721 |
| Driving_pressure | No-TPDP group | 0.984 | 0.000 | 4426 |
| Driving_pressure | TPDP group | 0.993 | 0.178 | 295 |
| Mechanical_power | All | 0.943 | 0.000 | 4721 |
| Mechanical_power | No-TPDP group | 0.947 | 0.000 | 4426 |
| Mechanical_power | TPDP group | 0.962 | 0.000 | 295 |
| Mechanical_ventilation_hour | All | 0.701 | 0.000 | 4721 |
| Mechanical_ventilation_hour | No-TPDP group | 0.689 | 0.000 | 4426 |
| Mechanical_ventilation_hour | TPDP group | 0.866 | 0.000 | 295 |

Normality test of variables in TPDP Group and No-TPDP Group in matched cohort.

| Variable | Test_Scope | W_statistic | P_value | N |
| --- | --- | --- | --- | --- |
| Age | All | 0.986 | 0.000 | 526 |
| Age | No-TPDP group | 0.987 | 0.014 | 263 |
| Age | TPDP group | 0.982 | 0.002 | 263 |
| BMI | All | 0.940 | 0.000 | 526 |
| BMI | No-TPDP group | 0.936 | 0.000 | 263 |
| BMI | TPDP group | 0.937 | 0.000 | 263 |
| SOFA_score | All | 0.986 | 0.000 | 526 |
| SOFA_score | No-TPDP group | 0.981 | 0.001 | 263 |
| SOFA_score | TPDP group | 0.984 | 0.004 | 263 |
| APSIII | All | 0.988 | 0.000 | 526 |
| APSIII | No-TPDP group | 0.982 | 0.002 | 263 |
| APSIII | TPDP group | 0.990 | 0.074 | 263 |
| ABPd | All | 0.985 | 0.000 | 526 |
| ABPd | No-TPDP group | 0.980 | 0.001 | 263 |
| ABPd | TPDP group | 0.987 | 0.021 | 263 |
| ABPs | All | 0.978 | 0.000 | 526 |
| ABPs | No-TPDP group | 0.984 | 0.005 | 263 |
| ABPs | TPDP group | 0.968 | 0.000 | 263 |
| Heart_Rate | All | 0.990 | 0.001 | 526 |
| Heart_Rate | No-TPDP group | 0.979 | 0.001 | 263 |
| Heart_Rate | TPDP group | 0.992 | 0.197 | 263 |
| WBC | All | 0.990 | 0.001 | 526 |
| WBC | No-TPDP group | 0.988 | 0.027 | 263 |
| WBC | TPDP group | 0.985 | 0.007 | 263 |
| Creatinine | All | 0.617 | 0.000 | 526 |
| Creatinine | No-TPDP group | 0.767 | 0.000 | 263 |
| Creatinine | TPDP group | 0.530 | 0.000 | 263 |
| Glucose | All | 0.981 | 0.000 | 526 |
| Glucose | No-TPDP group | 0.974 | 0.000 | 263 |
| Glucose | TPDP group | 0.982 | 0.002 | 263 |
| BUN | All | 0.916 | 0.000 | 526 |
| BUN | No-TPDP group | 0.918 | 0.000 | 263 |
| BUN | TPDP group | 0.912 | 0.000 | 263 |
| PT | All | 0.959 | 0.000 | 526 |
| PT | No-TPDP group | 0.962 | 0.000 | 263 |
| PT | TPDP group | 0.956 | 0.000 | 263 |
| PlateletCount | All | 0.966 | 0.000 | 526 |
| PlateletCount | No-TPDP group | 0.965 | 0.000 | 263 |
| PlateletCount | TPDP group | 0.963 | 0.000 | 263 |
| Lac | All | 0.949 | 0.000 | 526 |
| Lac | No-TPDP group | 0.955 | 0.000 | 263 |
| Lac | TPDP group | 0.934 | 0.000 | 263 |
| Arterial_PH | All | 0.847 | 0.000 | 526 |
| Arterial_PH | No-TPDP group | 0.858 | 0.000 | 263 |
| Arterial_PH | TPDP group | 0.833 | 0.000 | 263 |
| Arterial_O2_pressure | All | 0.952 | 0.000 | 526 |
| Arterial_O2_pressure | No-TPDP group | 0.952 | 0.000 | 263 |
| Arterial_O2_pressure | TPDP group | 0.947 | 0.000 | 263 |
| Arterial_CO2_Pressure | All | 0.993 | 0.010 | 526 |
| Arterial_CO2_Pressure | No-TPDP group | 0.992 | 0.138 | 263 |
| Arterial_CO2_Pressure | TPDP group | 0.991 | 0.096 | 263 |
| HCO3 | All | 0.986 | 0.000 | 526 |
| HCO3 | No-TPDP group | 0.984 | 0.005 | 263 |
| HCO3 | TPDP group | 0.986 | 0.010 | 263 |
| PFratio | All | 0.908 | 0.000 | 526 |
| PFratio | No-TPDP group | 0.901 | 0.000 | 263 |
| PFratio | TPDP group | 0.911 | 0.000 | 263 |
| Total_Respiratory_Rate | All | 0.986 | 0.000 | 526 |
| Total_Respiratory_Rate | No-TPDP group | 0.987 | 0.017 | 263 |
| Total_Respiratory_Rate | TPDP group | 0.981 | 0.001 | 263 |
| Spontaneous_Respiratory_Rate | All | 0.467 | 0.000 | 526 |
| Spontaneous_Respiratory_Rate | No-TPDP group | 0.480 | 0.000 | 263 |
| Spontaneous_Respiratory_Rate | TPDP group | 0.449 | 0.000 | 263 |
| Set_Respiratory_Rate | All | 0.978 | 0.000 | 526 |
| Set_Respiratory_Rate | No-TPDP group | 0.977 | 0.000 | 263 |
| Set_Respiratory_Rate | TPDP group | 0.974 | 0.000 | 263 |
| Tidal_Volume | All | 0.998 | 0.796 | 526 |
| Tidal_Volume | No-TPDP group | 0.988 | 0.024 | 263 |
| Tidal_Volume | TPDP group | 0.994 | 0.361 | 263 |
| PEEP | All | 0.936 | 0.000 | 526 |
| PEEP | No-TPDP group | 0.936 | 0.000 | 263 |
| PEEP | TPDP group | 0.937 | 0.000 | 263 |
| Plateau_Pressure | All | 0.992 | 0.009 | 526 |
| Plateau_Pressure | No-TPDP group | 0.989 | 0.046 | 263 |
| Plateau_Pressure | TPDP group | 0.991 | 0.106 | 263 |
| Peak_Pressure | All | 0.992 | 0.009 | 526 |
| Peak_Pressure | No-TPDP group | 0.986 | 0.010 | 263 |
| Peak_Pressure | TPDP group | 0.995 | 0.526 | 263 |
| Lung_compliance | All | 0.891 | 0.000 | 526 |
| Lung_compliance | No-TPDP group | 0.887 | 0.000 | 263 |
| Lung_compliance | TPDP group | 0.888 | 0.000 | 263 |
| Driving_pressure | All | 0.995 | 0.084 | 526 |
| Driving_pressure | No-TPDP group | 0.990 | 0.082 | 263 |
| Driving_pressure | TPDP group | 0.993 | 0.265 | 263 |
| Mechanical_power | All | 0.968 | 0.000 | 526 |
| Mechanical_power | No-TPDP group | 0.965 | 0.000 | 263 |
| Mechanical_power | TPDP group | 0.967 | 0.000 | 263 |
| Mechanical_ventilation_hour | All | 0.815 | 0.000 | 526 |
| Mechanical_ventilation_hour | No-TPDP group | 0.765 | 0.000 | 263 |
| Mechanical_ventilation_hour | TPDP group | 0.848 | 0.000 | 263 |

Normality test of variables in TPDP≤12.5cmH_2_O group and TPDP＞12.5cmH_2_O group.

| Variable | Test_Scope | W_statistic | P_value | N |
| --- | --- | --- | --- | --- |
| Age | All | 0.984 | 0.002 | 295 |
| Age | TPDP≤12.5cmH2O group | 0.981 | 0.003 | 231 |
| Age | TPDP＞12.5cmH2O group | 0.99 | 0.896 | 64 |
| BMI | All | 0.907 | 0.000 | 295 |
| BMI | TPDP≤12.5cmH2O group | 0.886 | 0.000 | 231 |
| BMI | TPDP＞12.5cmH2O group | 0.955 | 0.020 | 64 |
| SOFA_score | All | 0.988 | 0.012 | 295 |
| SOFA_score | TPDP≤12.5cmH2O group | 0.989 | 0.081 | 231 |
| SOFA_score | TPDP＞12.5cmH2O group | 0.972 | 0.150 | 64 |
| APSIII | All | 0.99 | 0.047 | 295 |
| APSIII | TPDP≤12.5cmH2O group | 0.991 | 0.172 | 231 |
| APSIII | TPDP＞12.5cmH2O group | 0.971 | 0.144 | 64 |
| ABPd | All | 0.987 | 0.008 | 295 |
| ABPd | TPDP≤12.5cmH2O group | 0.98 | 0.003 | 231 |
| ABPd | TPDP＞12.5cmH2O group | 0.984 | 0.565 | 64 |
| ABPs | All | 0.967 | 0.000 | 295 |
| ABPs | TPDP≤12.5cmH2O group | 0.962 | 0.000 | 231 |
| ABPs | TPDP＞12.5cmH2O group | 0.968 | 0.091 | 64 |
| Heart_Rate | All | 0.992 | 0.088 | 295 |
| Heart_Rate | TPDP≤12.5cmH2O group | 0.987 | 0.040 | 231 |
| Heart_Rate | TPDP＞12.5cmH2O group | 0.988 | 0.784 | 64 |
| WBC | All | 0.988 | 0.019 | 295 |
| WBC | TPDP≤12.5cmH2O group | 0.986 | 0.027 | 231 |
| WBC | TPDP＞12.5cmH2O group | 0.986 | 0.673 | 64 |
| Creatinine | All | 0.564 | 0.000 | 295 |
| Creatinine | TPDP≤12.5cmH2O group | 0.519 | 0.000 | 231 |
| Creatinine | TPDP＞12.5cmH2O group | 0.828 | 0.000 | 64 |
| Glucose | All | 0.982 | 0.001 | 295 |
| Glucose | TPDP≤12.5cmH2O group | 0.981 | 0.004 | 231 |
| Glucose | TPDP＞12.5cmH2O group | 0.972 | 0.159 | 64 |
| BUN | All | 0.916 | 0.000 | 295 |
| BUN | TPDP≤12.5cmH2O group | 0.906 | 0.000 | 231 |
| BUN | TPDP＞12.5cmH2O group | 0.927 | 0.001 | 64 |
| PT | All | 0.949 | 0.000 | 295 |
| PT | TPDP≤12.5cmH2O group | 0.945 | 0.000 | 231 |
| PT | TPDP＞12.5cmH2O group | 0.954 | 0.018 | 64 |
| PlateletCount | All | 0.967 | 0.000 | 295 |
| PlateletCount | TPDP≤12.5cmH2O group | 0.965 | 0.000 | 231 |
| PlateletCount | TPDP＞12.5cmH2O group | 0.97 | 0.125 | 64 |
| Lac | All | 0.936 | 0.000 | 295 |
| Lac | TPDP≤12.5cmH2O group | 0.941 | 0.000 | 231 |
| Lac | TPDP＞12.5cmH2O group | 0.91 | 0.000 | 64 |
| Arterial_PH | All | 0.836 | 0.000 | 295 |
| Arterial_PH | TPDP≤12.5cmH2O group | 0.832 | 0.000 | 231 |
| Arterial_PH | TPDP＞12.5cmH2O group | 0.83 | 0.000 | 64 |
| Arterial_O2_pressure | All | 0.951 | 0.000 | 295 |
| Arterial_O2_pressure | TPDP≤12.5cmH2O group | 0.95 | 0.000 | 231 |
| Arterial_O2_pressure | TPDP＞12.5cmH2O group | 0.935 | 0.002 | 64 |
| Arterial_CO2_Pressure | All | 0.99 | 0.049 | 295 |
| Arterial_CO2_Pressure | TPDP≤12.5cmH2O group | 0.993 | 0.388 | 231 |
| Arterial_CO2_Pressure | TPDP＞12.5cmH2O group | 0.966 | 0.078 | 64 |
| HCO3 | All | 0.988 | 0.017 | 295 |
| HCO3 | TPDP≤12.5cmH2O group | 0.987 | 0.029 | 231 |
| HCO3 | TPDP＞12.5cmH2O group | 0.986 | 0.661 | 64 |
| PFratio | All | 0.899 | 0.000 | 295 |
| PFratio | TPDP≤12.5cmH2O group | 0.899 | 0.000 | 231 |
| PFratio | TPDP＞12.5cmH2O group | 0.89 | 0.000 | 64 |
| Total_Respiratory_Rate | All | 0.978 | 0.000 | 295 |
| Total_Respiratory_Rate | TPDP≤12.5cmH2O group | 0.978 | 0.001 | 231 |
| Total_Respiratory_Rate | TPDP＞12.5cmH2O group | 0.958 | 0.029 | 64 |
| Spontaneous_Respiratory_Rate | All | 0.427 | 0.000 | 295 |
| Spontaneous_Respiratory_Rate | TPDP≤12.5cmH2O group | 0.453 | 0.000 | 231 |
| Spontaneous_Respiratory_Rate | TPDP＞12.5cmH2O group | 0.364 | 0.000 | 64 |
| Set_Respiratory_Rate | All | 0.966 | 0.000 | 295 |
| Set_Respiratory_Rate | TPDP≤12.5cmH2O group | 0.967 | 0.000 | 231 |
| Set_Respiratory_Rate | TPDP＞12.5cmH2O group | 0.956 | 0.024 | 64 |
| Tidal_Volume | All | 0.996 | 0.571 | 295 |
| Tidal_Volume | TPDP≤12.5cmH2O group | 0.991 | 0.195 | 231 |
| Tidal_Volume | TPDP＞12.5cmH2O group | 0.987 | 0.753 | 64 |
| PEEP | All | 0.889 | 0.000 | 295 |
| PEEP | TPDP≤12.5cmH2O group | 0.903 | 0.000 | 231 |
| PEEP | TPDP＞12.5cmH2O group | 0.862 | 0.000 | 64 |
| Plateau_Pressure | All | 0.987 | 0.009 | 295 |
| Plateau_Pressure | TPDP≤12.5cmH2O group | 0.988 | 0.050 | 231 |
| Plateau_Pressure | TPDP＞12.5cmH2O group | 0.952 | 0.014 | 64 |
| Peak_Pressure | All | 0.991 | 0.069 | 295 |
| Peak_Pressure | TPDP≤12.5cmH2O group | 0.993 | 0.302 | 231 |
| Peak_Pressure | TPDP＞12.5cmH2O group | 0.97 | 0.122 | 64 |
| Lung_compliance | All | 0.881 | 0.000 | 295 |
| Lung_compliance | TPDP≤12.5cmH2O group | 0.874 | 0.000 | 231 |
| Lung_compliance | TPDP＞12.5cmH2O group | 0.92 | 0.000 | 64 |
| Driving_pressure | All | 0.993 | 0.178 | 295 |
| Driving_pressure | TPDP≤12.5cmH2O group | 0.989 | 0.073 | 231 |
| Driving_pressure | TPDP＞12.5cmH2O group | 0.992 | 0.941 | 64 |
| Mechanical_power | All | 0.962 | 0.000 | 295 |
| Mechanical_power | TPDP≤12.5cmH2O group | 0.958 | 0.000 | 231 |
| Mechanical_power | TPDP＞12.5cmH2O group | 0.97 | 0.116 | 64 |
| Mechanical_ventilation_hour | All | 0.866 | 0.000 | 295 |
| Mechanical_ventilation_hour | TPDP≤12.5cmH2O group | 0.859 | 0.000 | 231 |
| Mechanical_ventilation_hour | TPDP＞12.5cmH2O group | 0.893 | 0.000 | 64 |
