# Supplementary material for: The effect of target transpulmonary driving pressure values on mortality in ARDS patients: A retrospective study based on the MIMIC-IV database
Source: PLoS One. 2025 Jun 18;20(6):e0326060. doi: 10.1371/journal.pone.0326060 (PMC12176163; doi:10.1371/journal.pone.0326060)
Supplement: S2 Table — (DOCX) [file pone.0326060.s011.docx]

**eTable 2** The association between TPDP levels and mortality.

The association between TPDP levels and 28-day mortality.

| Categories | OR | lower .95 | upper .95 | *P*-value |
| --- | --- | --- | --- | --- |
| P/F ratio≤100mmHg | 1.163 | 1.065 | 1.270 | 0.001 |
| 100mmHg<P/F ratio≤200mmHg | 1.213 | 1.079 | 1.363 | 0.001 |
| 200mmHg<P/F ratio≤300mmHg | 1.276 | 0.960 | 1.697 | 0.093 |

The association between TPDP levels and Hospital mortality.

| Categories | OR | lower .95 | upper .95 | *P*-value |
| --- | --- | --- | --- | --- |
| P/F ratio≤100mmHg | 1.112 | 1.023 | 1.210 | 0.013 |
| 100mmHg<P/F ratio≤200mmHg | 1.208 | 1.076 | 1.357 | 0.001 |
| 200mmHg<P/F ratio≤300mmHg | 1.217 | 0.940 | 1.575 | 0.137 |

The association between TPDP levels and ICU mortality.

| Categories | OR | lower .95 | upper .95 | *P*-value |
| --- | --- | --- | --- | --- |
| P/F ratio≤100mmHg | 1.129 | 1.037 | 1.231 | 0.005 |
| 100mmHg<P/F ratio≤200mmHg | 1.198 | 1.068 | 1.343 | 0.002 |
| 200mmHg<P/F ratio≤300mmHg | 1.115 | 0.855 | 1.454 | 0.421 |
