# Supplementary material for: The effect of target transpulmonary driving pressure values on mortality in ARDS patients: A retrospective study based on the MIMIC-IV database
Source: PLoS One. 2025 Jun 18;20(6):e0326060. doi: 10.1371/journal.pone.0326060 (PMC12176163; doi:10.1371/journal.pone.0326060)
Supplement: S3 Table — (DOCX) [file pone.0326060.s012.docx]

**eTable 3** The comparison of laboratory test and outcomes between TPDP group and No-TPDP group.

|  | **Original Cohort** | | | **Matched Cohort** | | |
| --- | --- | --- | --- | --- | --- | --- |
|  | **TPDP group** | **No-TPDP group** | ***P*** | **TPDP group** | **No-TPDP group** | ***P*** |
| **n** | **295** | **4426** |  | **263** | **263** |  |
| **Laboratory test** |  |  |  |  |  |  |
| WBC (median [IQR]) | 12.10 [8.30, 16.20] | 11.80 [8.60, 15.60] | 0.505 | 12.00 [8.05, 15.80] | 11.60 [7.65, 15.65] | 0.389 |
| Glucose (median [IQR]) | 137.00 [115.40, 166.95] | 132.75 [110.50, 156.50] | 0.009 | 135.50 [113.15, 164.75] | 139.50 [116.90, 158.75] | 0.556 |
| Creatinine (median [IQR]) | 1.30 [0.90, 2.30] | 1.10 [0.70, 1.80] | <0.001 | 1.30 [0.90, 2.20] | 1.20 [0.80, 2.10] | 0.366 |
| BUN (median [IQR]) | 24.80 [16.80, 36.55] | 22.00 [14.50, 35.00] | 0.015 | 24.00 [16.30, 35.60] | 23.00 [15.00, 35.00] | 0.499 |
| Lac (median [IQR]) | 2.20 [1.40, 3.30] | 1.90 [1.30, 2.70] | <0.001 | 2.20 [1.40, 3.30] | 2.20 [1.60, 3.35] | 0.446 |
| Platelet Count (median [IQR]) | 166.00 [110.15, 237.25] | 171.00 [109.50, 235.45] | 0.845 | 165.00 [107.50, 230.90] | 162.80 [95.65, 228.15] | 0.518 |
| PT(median [IQR]) | 14.80 [13.35, 17.55] | 14.40 [13.00, 16.60] | 0.009 | 14.90 [13.45, 17.70] | 15.10 [13.35, 17.80] | 0.658 |
| **Outcomes** |  |  |  |  |  |  |
| Mechanical ventilation hour (median [IQR]) | 123.10 [72.15, 195.00] | 60.00 [37.00, 103.90] | <0.001 | 118.80 [67.90, 188.50] | 82.10 [55.20, 149.20] | <0.001 |
| ICU staydays (median [IQR]) | 10.60 [5.75, 19.05] | 6.70 [3.70, 12.30] | <0.001 | 10.50 [5.70, 19.05] | 8.40 [4.10, 13.75] | 0.002 |
| Hospital staydays(median [IQR]) | 15.90 [8.05, 26.60] | 12.00 [6.40, 20.60] | <0.001 | 15.80 [8.20, 26.95] | 12.00 [5.15, 21.55] | 0.001 |
| 28-day survival day(median [IQR]) | 28.00 [10.65, 28.00] | 28.00 [13.30, 28.00] | 0.043 | 28.00 [11.15, 28.00] | 28.00 [5.70, 28.00] | 0.118 |
| 28-day mortality (%) | 107 (36.27) | 1361 (30.75) | 0.047 | 95 (36.12) | 111 (42.21) | 0.153 |
| Hospital mortality(%) | 114 (38.64) | 1305 (29.48) | 0.001 | 103 (39.16) | 108 (41.06) | 0.656 |
| ICU mortality (%) | 102 (34.58) | 1116 (25.21) | <0.001 | 91 (34.60) | 110 (41.83) | 0.088 |
