# Supplementary material for: The effect of target transpulmonary driving pressure values on mortality in ARDS patients: A retrospective study based on the MIMIC-IV database
Source: PLoS One. 2025 Jun 18;20(6):e0326060. doi: 10.1371/journal.pone.0326060 (PMC12176163; doi:10.1371/journal.pone.0326060)
Supplement: S4 Table — (DOCX) [file pone.0326060.s013.docx]

**eTable 4** The comparison of Demographic characteristics, The severity of illness, Vital signs, and Laboratory test between TPDP > 12.5cmH_2_O group and TPDP ≤ 12.5cmH_2_O group.

|  | **TPDP > 12.5cmH_2_O** | **TPDP ≤ 12.5cmH_2_O** | ***P*** |
| --- | --- | --- | --- |
| **n** | **64** | **231** |  |
| **Demographic characteristics** |  |  |  |
| Age (median [IQR]) | 56.70 [47.75, 67.20] | 57.90 [43.55, 66.80] | 0.836 |
| BMI (median [IQR]) | 31.55 [25.45, 37.50] | 31.20 [26.90, 36.40] | 0.626 |
| **The severity of illness** |  |  |  |
| APSIII (median [IQR]) | 99.25 (31.51) | 88.46 (30.11) | 0.013 |
| SOFA score (median [IQR]) | 9.69 (3.23) | 9.51 (3.25) | 0.697 |
| **Vital signs** |  |  |  |
| ABPd (median [IQR]) | 57.95 [52.92, 62.50] | 58.30 [54.60, 62.25] | 0.500 |
| ABPs (median [IQR]) | 107.40 [102.62, 113.90] | 107.40 [102.00, 112.35] | 0.552 |
| Heart Rate (median [IQR]) | 95.65 [86.80, 105.18] | 93.70 [81.15, 106.05] | 0.208 |
| **Laboratory test** |  |  |  |
| WBC (median [IQR]) | 12.25 [9.07, 16.25] | 12.10 [7.95, 16.20] | 0.482 |
| Glucose (median [IQR]) | 134.35 [113.93, 180.12] | 137.40 [116.00, 163.80] | 0.677 |
| Creatinine (median [IQR]) | 1.30 [0.90, 2.62] | 1.30 [0.90, 2.20] | 0.718 |
| BUN (median [IQR]) | 28.60 [19.23, 39.48] | 23.70 [16.30, 35.20] | 0.085 |
| Lac (median [IQR]) | 1.70 [1.40, 3.10] | 2.40 [1.40, 3.35] | 0.292 |
| Platelet Count (median [IQR]) | 158.40 [98.75, 219.68] | 167.00 [110.35, 243.15] | 0.321 |
| PT(median [IQR]) | 15.05 [13.50, 17.47] | 14.80 [13.25, 17.55] | 0.834 |
