# Supplementary material for: The effect of target transpulmonary driving pressure values on mortality in ARDS patients: A retrospective study based on the MIMIC-IV database
Source: PLoS One. 2025 Jun 18;20(6):e0326060. doi: 10.1371/journal.pone.0326060 (PMC12176163; doi:10.1371/journal.pone.0326060)
Supplement: S5 Table — (DOCX) [file pone.0326060.s014.docx]

**eTable 5** Characteristics of the three phenotypes.

|  | **Overall** | **Phenotype-I** | **Phenotype-II** | **Phenotype-III** | ***P*** |
| --- | --- | --- | --- | --- | --- |
| **n** | **295** | **138** | **62** | **95** |  |
| **Demographic characteristics** |  |  |  |  |  |
| Age (median [IQR]) | 57.60 [43.85, 66.90] | 59.70 [42.18, 68.55] | 55.65 [44.52, 62.88] | 57.30 [44.80, 66.35] | 0.348 |
| BMI (median [IQR]) | 31.40 [26.65, 36.75] | 31.75 [26.13, 38.70] | 31.15 [27.70, 35.83] | 30.30 [26.30, 35.80] | 0.450 |
| **The severity of illness** |  |  |  |  |  |
| APSIII (median [IQR]) | 91.00 [66.00, 112.50] | 93.50 [64.00, 114.00] | 88.00 [66.00, 105.75] | 90.00 [68.50, 113.00] | 0.710 |
| SOFA score (median [IQR]) | 9.20 [7.25, 11.60] | 9.15 [7.20, 12.00] | 9.40 [7.03, 11.60] | 9.40 [7.75, 11.40] | 0.838 |
| **Vital signs** |  |  |  |  |  |
| ABPd (median [IQR]) | 58.10 [54.25, 62.30] | 58.25 [53.85, 62.38] | 59.05 [54.92, 63.35] | 57.60 [54.55, 61.65] | 0.345 |
| ABPs (median [IQR]) | 107.40 [102.10, 112.60] | 106.20 [101.35, 112.35] | 108.20 [103.60, 112.97] | 108.50 [103.70, 112.65] | 0.217 |
| Heart Rate (median [IQR]) | 94.00 [82.25, 106.00] | 95.05 [83.10, 105.82] | 100.65 [80.03, 108.85] | 91.40 [82.75, 101.65] | 0.412 |
| **Laboratory test** |  |  |  |  |  |
| WBC (mean (SD)) | 12.51 (5.60) | 12.70 (5.95) | 12.45 (5.60) | 12.27 (5.10) | 0.842 |
| Glucose (median [IQR]) | 137.00 [115.40, 166.95] | 142.25 [111.03, 181.88] | 132.60 [118.00, 162.50] | 133.20 [117.10, 155.25] | 0.269 |
| Creatinine (median [IQR]) | 1.30 [0.90, 2.30] | 1.30 [0.92, 2.40] | 1.60 [1.00, 2.58] | 1.20 [0.80, 1.80] | 0.018 |
| BUN (median [IQR]) | 24.80 [16.80, 36.55] | 26.65 [17.77, 38.38] | 25.05 [17.50, 33.00] | 21.70 [14.25, 37.15] | 0.284 |
| Lac (median [IQR]) | 2.20 [1.40, 3.30] | 2.30 [1.40, 3.30] | 2.20 [1.50, 3.27] | 2.20 [1.40, 3.35] | 0.968 |
| Platelet Count (median [IQR]) | 166.00 [110.15, 237.25] | 175.15 [110.15, 234.15] | 157.75 [113.03, 217.07] | 166.50 [106.05, 270.50] | 0.734 |
| PT (median [IQR]) | 14.80 [13.35, 17.55] | 15.00 [13.50, 17.78] | 14.65 [13.03, 17.85] | 14.90 [13.50, 16.95] | 0.793 |
| Blood gas analysis parameters |  |  |  |  |  |
| Arterial PH (median [IQR]) | 7.30 [7.30, 7.40] | 7.30 [7.30, 7.40] | 7.30 [7.30, 7.40] | 7.30 [7.30, 7.40] | 0.804 |
| Arterial O_2_ pressure (median [IQR]) | 92.60 [80.95, 108.75] | 93.55 [83.35, 110.55] | 93.65 [77.50, 113.28] | 91.20 [80.50, 100.35] | 0.226 |
| Arterial CO_2_ Pressure (median [IQR]) | 43.80 [38.60, 49.20] | 44.25 [39.65, 49.00] | 41.95 [37.55, 48.65] | 43.80 [38.05, 51.25] | 0.342 |
| HCO3 (median [IQR]) | 21.00 [17.90, 24.50] | 21.90 [18.40, 24.60] | 20.60 [17.85, 23.00] | 20.50 [17.45, 24.55] | 0.431 |
| PF ratio (median [IQR]) | 95.00 [68.80, 136.90] | 98.45 [71.25, 135.32] | 98.00 [73.50, 142.02] | 85.00 [66.50, 134.75] | 0.592 |
| **Respiratory characteristics** |  |  |  |  |  |
| Tidal Volume (median [IQR]) | 427.30 [366.90, 474.75] | 436.55 [414.22, 457.82] | 527.40 [507.70, 556.18] | 346.60 [320.65, 364.35] | <0.001 |
| Spontaneous Respiratory Rate (median [IQR]) | 0.00 [0.00, 1.00] | 0.00 [0.00, 0.85] | 0.75 [0.00, 2.60] | 0.00 [0.00, 0.15] | <0.001 |
| Total Respiratory Rate (median [IQR]) | 26.30 [22.70, 29.25] | 25.90 [22.15, 29.25] | 25.65 [21.47, 27.98] | 27.70 [24.65, 29.95] | 0.015 |
| Set Respiratory Rate (median [IQR]) | 25.30 [21.80, 28.00] | 24.70 [21.65, 28.25] | 23.80 [19.85, 26.92] | 26.00 [23.45, 28.40] | 0.006 |
| PEEP (median [IQR]) | 10.50 [9.65, 12.00] | 10.80 [9.93, 12.00] | 10.25 [9.03, 12.00] | 10.30 [9.90, 12.00] | 0.435 |
| Plateau Pressure (mean (SD)) | 26.59 (4.22) | 26.69 (4.24) | 25.57 (4.32) | 27.11 (4.04) | 0.078 |
| Peak Pressure (mean (SD)) | 30.36 (5.15) | 30.58 (5.39) | 29.57 (5.69) | 30.56 (4.38) | 0.399 |
| Driving pressure (median [IQR]) | 15.30 [12.50, 18.65] | 15.25 [12.60, 17.70] | 14.70 [11.50, 16.65] | 16.30 [12.90, 19.50] | 0.070 |
|  | **Overall** | **Phenotype-I** | **Phenotype-II** | **Phenotype-III** | ***P*** |
| **n** | **295** | **138** | **62** | **95** |  |
| Mechanical power (median [IQR]) | 23.50 [18.55, 28.90] | 24.85 [19.02, 30.50] | 27.35 [22.10, 37.22] | 20.10 [17.00, 23.85] | <0.001 |
| Lung compliance (median [IQR]) | 27.50 [21.85, 34.90] | 28.65 [24.33, 34.68] | 36.95 [31.40, 46.88] | 20.80 [17.70, 25.95] | <0.001 |
| **Outcomes** |  |  |  |  |  |
| Mechanical ventilation hour (median [IQR]) | 123.10 [72.15, 195.00] | 119.05 [68.83, 188.73] | 119.05 [57.50, 183.98] | 135.60 [82.35, 227.50] | 0.217 |
| ICU stayday (median [IQR]) | 10.60 [5.75, 19.05] | 9.60 [5.62, 18.03] | 10.15 [6.60, 21.25] | 12.10 [5.35, 18.65] | 0.654 |
| Hospital stayday (median [IQR]) | 15.90 [8.05, 26.60] | 15.10 [7.15, 27.45] | 16.75 [7.85, 28.25] | 16.80 [9.70, 25.00] | 0.932 |
| 28d survival day (median [IQR]) | 28.00 [10.65, 28.00] | 28.00 [8.12, 28.00] | 28.00 [13.85, 28.00] | 28.00 [12.45, 28.00] | 0.637 |
| 28d mortality (%) | 107 (36.27) | 52 (37.68) | 19 (30.65) | 36 (37.89) | 0.584 |
| Hospital mortality (%) | 114 (38.64) | 56 (40.58) | 20 (32.26) | 38 (40.00) | 0.507 |
| ICU mortality (%) | 102 (34.58) | 51 (36.96) | 17 (27.42) | 34 (35.79) | 0.404 |
