# Supplementary material for: The effect of target transpulmonary driving pressure values on mortality in ARDS patients: A retrospective study based on the MIMIC-IV database
Source: PLoS One. 2025 Jun 18;20(6):e0326060. doi: 10.1371/journal.pone.0326060 (PMC12176163; doi:10.1371/journal.pone.0326060)
Supplement: Attachment — Submitted filename: Response to Reviewers.docx [file pone.0326060.s017.docx]

Dear Academic Editor and Reviewer,

We appreciate the insightful feedback on our manuscript and have revised the paper accordingly. Below, we outline our responses and corresponding changes:

1. Title and Content Revision:

We have revised the title and introductory sections to clarify the study's focus on target transpulmonary driving pressure (TPDP) values rather than the measurement of TPDP alone. This change aligns the title and text with the study's emphasis on target pressure values as the key influencing factors for ARDS outcomes (Page 1 lines1-4).

2. Incorporating ARDS Severity-Based Analysis:

Based on the reviewer’s recommendation, we have now included an analysis of ARDS severity categories according to both the Berlin Definition and the 2024 New Global Definition (mild, moderate, and severe) as indicators of patient prognosis. This stratified analysis is now reflected in the Results section (Page 6-7, lines 228-248), providing a clearer perspective on the impact of target TPDP values within distinct ARDS severity groups.

3. Clarifying TPDP Measurement in the Methods Section:

We expanded the Methods section to clarify the measurement approach for TPDP, differentiating it from conventional driving pressure. We specify that TDP was measured as the difference between end-inspiratory and end-expiratory transpulmonary pressures (Page 4, lines 140-144).

4. Simplifying the Results Section:

We streamlined the Results by focusing on essential findings and referring readers to the Tables and Figures for detailed data. We also reorganized extensive tables into smaller, logically grouped collections to improve readability and make the data easier to interpret (Page 7-9, lines 249-280).

5. Reorganizing and Condensing the Discussion:

The Discussion section was restructured to begin with a summary of key findings and the clinical implications of TPDP. We addressed whether we recommend TPDP monitoring for all ICU settings or selectively for specific ARDS phenotypes, and briefly mentioned the validation plans for the phenotypes identified. We also rephrased parts of the Discussion to interpret our findings within the context of current clinical practice, focusing on the study’s direct relevance to ICU management (Page 11-12, lines 325-386).

6. Clarifying Data Optimization and Analysis:

In response to the reviewer’s comments, we added details on the data optimization process, including the propensity score matching (PSM) approach and any limitations that remain unaddressed by PSM. We condensed the discussion of database-related details to focus on data analysis and methods for addressing limitations (Page 12, lines 370-377).

7. Comparison with Existing Literature:

We highlighted how this study contributes to the body of research on TPDP and TPDP, explaining how our findings align with or differ from prior studies. (Page 12, lines 378-386).

These revisions align our study more closely with the reviewer’s recommendations, enhancing both clarity and clinical relevance. Thank you for the valuable suggestions, and we look forward to your feedback on the revised manuscript.
