# Supplementary material for: The effect of target transpulmonary driving pressure values on mortality in ARDS patients: A retrospective study based on the MIMIC-IV database
Source: PLoS One. 2025 Jun 18;20(6):e0326060. doi: 10.1371/journal.pone.0326060 (PMC12176163; doi:10.1371/journal.pone.0326060)
Supplement: Attachment — Submitted filename: PLOS ONE ARDSR1.docx [file pone.0326060.s016.docx]

The authors have done an excellent job of addressing the reviewer’s queries. However, there is still an underlying limitation of this study that needs to be fully addressed. The authors’ underlying assumption seems to be that the treatment of patients who had transpulmonary pressures measured somehow differed from those where pressures were not measured. The mediation analysis is helpful but still falls short of explaining the findings of the study. The authors should present data about the clinical interventions used in each patient to reduce TPP.

The authors unfortunately cannot assume that all study patients from the database received similar or any interventions based on TPP. The clinician reader will still not know how to apply the findings from this study in their patient population. Because it is unlikely that the act of measuring TPP decreases mortality, the reader needs to know what interventions they should perform to achieve a specific set of TPP values associated with better outcome.

The authors used a mediation analysis to suggest a possible common factor {intervention). While this analysis produced a compelling finding, the reader does not know if the same intervention was taken in all patients, or if any intervention was taken in all members of the TPP-study group. It is possible that various or no interventions were used. This limits the clinical application of the findings. An explanation of causation is necessary to support the association. Statistical associations can be accidental and rigorous relationships between two variables need an explanation of cause. Even then, additional testing is necessary.

It seems unlikely the authors can present information showing that patients who had TPP measured were given similar interventions. The Discussion must then include a paragraph that describes in detail this important shortcoming and how this limits interpretation of the data. The authors should also include a future plan on how to evaluate their hypothesis that TPP measurements are linked to interventions that reduce mortality.

Page 2, line 77

Could the authors please use a more descriptive word than “traditional.” The latter word has different meanings to various readers and is not scientifically descriptive.

Page 3, Line 83

The following sentence is not clear. Please clarify: “However, the heterogeneity of disease manifestation among ARDS patients is ignored when only using lower tidal volume ventilation calculated by ideal body weight or limited platform pressure.” Do the authors mean that the current lung protective strategies do not take disease heterogeneity into account? And, what do the authors mean by disease heterogeneity? Severity, etiology or other?

Page 3, Line 87

A recent meta-analysis conducted through a randomized control trial by the authors found that mechanical ventilation 88 guided by TPP was associated with decreased mortality among patients with ARDS.

Can the authors please explain how they conducted a meta-analysis within a randomized controlled trial? This does not make sense. The two research designs are entirely different. Please clarify.

Page 3, Line 90:

Please reword the following to clarify the concept: Pirrone’s study found that end-expiratory transpulmonary pressure above 0 cm 90 H2O promoted collapsed alveolar reopen

Page 3, Line 95

Please provide a reference for this statement: However, excessive transpulmonary pressure may lead to alveolar overdistension, raising the occurrence of VILI.

Can the authors please include the tests used to determine distribution of the data (e.g., normal vs skewed)?

Can the authors please provide more information about “spontaneous respiration” under propensity matching variables? Is this over breathing the set respiratory rate or something else?

The Discussion still needs work to have a clear presentation.

The Discussion is a literary forum that is used to interpret your findings and put your data in context of the existing literature. This Discussion reads more like a review of the literature rather than a scientific discussion of the study.

Please:

!) start your Discussion with the paragraph starting at line 412 on page 13.

2) Then please discuss the pros and cons of your data source: using a database for your analysis as opposed to performing a clinical trial. Consider the reliability of the databank and the type of information. While this database was rich in patient variables, it was not designed to support an analysis of outcomes for etiology. Therefore, it lacks details that are needed for direct correlation analysis. This explains why your study lacks information on causation and why you needed a substitute mediation analysis. However, as pointed out a medication analysis is still statistical conjecture that requires further testing. Further, it lacks practical description needed by clinicians for patient application.

3) Discuss the internal and external validity of your findings based on the methods you used. This will require a critical appraisal of your statistical methods. You can point out that your data source is from multiple institutions and therefore can be generalized to other practice sites with similar resources and facilities to those that participated in the data base. It should be noted that these findings may not be applicable to clinical practice centers that differ from those in the data source.

4) Summarize the overall limitations of this study and what has to be done to overcome the issues. (For example, you could say that while statistical analysis of the database provided preliminary evidence that careful control of TPP could improve ARDS outcomes, controlled clinical trials are still necessary to identify all confounders in outcomes and the effects of various interventions taken to achieve a TPP of 12.5 cm or less.

5) Provide precise conclusions based on the entirety of the Discussion.

6) Please remove all descriptive information in the Discussion unless it is needed to support a statement in the 5 major sections described above. Right now, your Discussion is closer to a review and fails to interpret your data.
