# Supplementary material for: The effect of target transpulmonary driving pressure values on mortality in ARDS patients: A retrospective study based on the MIMIC-IV database
Source: PLoS One. 2025 Jun 18;20(6):e0326060. doi: 10.1371/journal.pone.0326060 (PMC12176163; doi:10.1371/journal.pone.0326060)
Supplement: Attachment — Submitted filename: PLOSONEPoint-by-point response.docx [file pone.0326060.s018.docx]

Dear Editors and Reviewers:

Thank you for your letter and for the Reviewers’ comments concerning our manuscript entitled “The Effect of Target Transpulmonary Driving Pressure Values on Mortality in ARDS Patients: A Retrospective Study Based on the MIMIC-IV Database”. (Submission ID PONE-D-24-38166R1). Those comments are all valuable and very helpful for revising and improving our paper, as well as the important guiding significance to our researches. We have studied comments carefully and have made correction which we hope meet with approval. Revised portion are marked in red in the paper. The main corrections in the paper and the responds to the reviewer's comments are as following:

**Responses to the Reviewer 1’s comments:**

1. **Reviewer’ s comment: The reviewer pointed out a limitation related to the assumption that patients with measured transpulmonary pressures (TPDP) might have received different treatments compared to those without TPDP measurements. The reviewer requested more information on the clinical interventions used to reduce TPDP and emphasized that presenting data on interventions is critical for clinical application.**

**Response:** In response to the reviewer’s concern, we found that patients with decreased TPDP had a significantly lower mortality rate. It is important to emphasize that TPDP target is primarily a method for assessing respiratory mechanics rather than an intervention that directly impacts patient prognosis. We have revised the title and results sections to clarify the study's focus on target transpulmonary driving pressure (TPDP) values rather than the measurement of TPDP alone. This change aligns the title and text with the study's emphasis on target pressure values as the key influencing factors for ARDS outcomes. To investigate why patients with TPDP target had lower mortality, we examined the mortality differences and found that a TPDP cutoff value of 12.5 cmH2O was significant. Based on this finding, we grouped all patients who had TPDP target and observed that patients with TPDP < 12.5 cmH2O had a lower mortality rate. Notably, 78.3% of the patients in the TPDP-monitored group had TPDP values < =12.5 cmH2O, which accounted for a substantial proportion of the cohort. This suggests that the key factor contributing to the lower mortality in the TPDP-monitored group is effectively controlling TPDP below 12.5 cmH2O. We further investigated the specific parameters that could help achieve and maintain TPDP <= 12.5 cmH2O and found that effective control of peak airway pressure, plateau pressure, and driving pressure were critical in maintaining optimal TPDP values. Our mediation analysis also confirmed that peak airway pressure is an important mediator between TPDP > 12.5 cmH2O and increased mortality. To further substantiate these findings, we performed a separate analysis using propensity score matching (PSM) on patients with TPDP <= 12.5 cmH2O. After matching these patients to No-TPDP group with similar baseline characteristics, we observed that effective control of TPDP resulted in a significant reduction in mortality. In summary, while TPDP target serves as a method for assessing respiratory mechanics, the key to improving mortality outcomes lies in effectively controlling TPDP < 12.5 cmH2O. We hope these revisions enhance the clarity of the clinical implications of our findings. Thank you again for your thoughtful feedback. Please let us know if further clarifications are needed.

**Table 1. The comparison between No-TPDP group and TPDP ≤ 12.5cmH2O group in matched cohort.**

|  | No-TPDP | TPDP<=12.5cmH2O | *p* |
| --- | --- | --- | --- |
| n | 226 | 226 |  |
| Age (median [IQR]) | 58.35 [48.02, 68.80] | 58.25 [43.12, 66.95] | 0.497 |
| BMI (median [IQR]) | 31.40 [28.10, 36.80] | 31.05 [26.90, 36.27] | 0.372 |
| APSIII (median [IQR]) | 88.00 [63.00, 116.75] | 88.00 [65.00, 106.00] | 0.496 |
| SOFA_score (median [IQR]) | 8.95 [7.00, 11.78] | 9.25 [7.03, 11.60] | 0.833 |
| ABPd (median [IQR]) | 57.80 [54.45, 62.10] | 58.15 [54.52, 62.18] | 0.830 |
| ABPs (median [IQR]) | 108.45 [102.60, 114.72] | 107.55 [101.95, 112.42] | 0.329 |
| Heart_Rate (median [IQR]) | 92.40 [79.90, 105.00] | 93.05 [80.32, 105.30] | 0.810 |
| WBC (median [IQR]) | 12.95 [9.20, 16.35] | 12.00 [7.93, 16.10] | 0.213 |
| Glucose (median [IQR]) | 138.65 [111.70, 158.00] | 137.15 [115.20, 163.65] | 0.561 |
| Creatinine (median [IQR]) | 1.45 [0.90, 2.30] | 1.30 [0.90, 2.18] | 0.821 |
| BUN (median [IQR]) | 26.00 [17.85, 39.00] | 23.25 [16.08, 35.25] | 0.065 |
| Lac (median [IQR]) | 2.15 [1.60, 3.30] | 2.25 [1.40, 3.38] | 0.941 |
| PlateletCount (median [IQR]) | 178.20 [107.30, 246.25] | 166.25 [110.32, 242.25] | 0.672 |
| PT (median [IQR]) | 14.90 [13.50, 17.48] | 14.75 [13.33, 17.62] | 0.645 |
| Arterial_PH (median [IQR]) | 7.30 [7.30, 7.40] | 7.30 [7.30, 7.40] | 0.189 |
| Arterial_O2_pressure (median [IQR]) | 94.30 [79.80, 108.30] | 93.30 [82.22, 108.52] | 0.895 |
| Arterial_CO2_Pressure (median [IQR]) | 42.20 [37.00, 48.25] | 42.75 [38.00, 48.10] | 0.705 |
| HCO3 (median [IQR]) | 21.00 [18.00, 24.15] | 20.80 [17.80, 23.95] | 0.643 |
| PFratio (median [IQR]) | 91.85 [70.65, 133.72] | 95.10 [70.17, 142.00] | 0.694 |
| Tidal_Volume (median [IQR]) | 460.75 [409.52, 520.50] | 427.20 [367.95, 474.82] | <0.001 |
| Spontaneous_Respiratory_Rate (median [IQR]) | 0.30 [0.00, 3.08] | 0.00 [0.00, 1.17] | <0.001 |
| Total_Respiratory_Rate (median [IQR]) | 24.35 [20.30, 27.78] | 26.05 [22.60, 28.87] | 0.001 |
| Set_Respiratory_Rate (median [IQR]) | 22.70 [18.70, 26.00] | 25.30 [21.60, 28.00] | <0.001 |
| PEEP (median [IQR]) | 9.50 [7.20, 10.60] | 10.60 [9.53, 12.00] | <0.001 |
| Plateau_Pressure (median [IQR]) | 23.10 [19.80, 26.70] | 26.35 [23.30, 29.08] | <0.001 |
| Peak_Pressure (median [IQR]) | 26.35 [22.72, 30.87] | 30.30 [26.22, 33.40] | <0.001 |
| Lung_compliance (median [IQR]) | 33.75 [25.58, 43.08] | 28.35 [22.63, 36.10] | <0.001 |
| Driving_pressure (median [IQR]) | 13.75 [11.30, 16.17] | 14.95 [12.10, 18.30] | 0.002 |
| Mechanical_power (median [IQR]) | 20.35 [15.30, 26.58] | 23.10 [18.30, 28.25] | 0.002 |
| Mechanical_ventilation_hour (median [IQR]) | 83.05 [47.85, 141.62] | 119.30 [71.72, 184.10] | <0.001 |
| ICUstayday (median [IQR]) | 7.65 [3.80, 13.88] | 10.70 [6.10, 19.62] | <0.001 |
| Hospitalstayday (median [IQR]) | 13.15 [4.90, 21.20] | 17.50 [8.83, 27.50] | <0.001 |
| 28d_survival_day (median [IQR]) | 28.00 [6.88, 28.00] | 28.00 [16.55, 28.00] | 0.029 |
| 28d_mortality(%) | 87 (38.50) | 66 (29.20) | 0.037 |
| ICUmortality (%) | 84 (37.17) | 64 (28.32) | 0.045 |

2. **Reviewer’ s comment: Page 2, Line 77: Use of the word "traditional"**

**Response:** Thank you for the reviewer’s insightful comment. You are absolutely correct, and we acknowledge that we made a fault in referring to the "ARDSnet low tidal volume and plateau pressure control ventilation strategy from 2004" as the "traditional" lung-protective ventilation strategy. As you pointed out, this strategy differs significantly from the "traditional mechanical ventilation strategy," particularly with regard to the control of tidal volume and plateau pressure. We have revised the manuscript accordingly. We no longer refer to the 2004 ARDSnet strategy as "traditional." Instead, we now refer to it as the "lung-protective ventilation strategy (LPVS)." This change has been made in the revised manuscript, and we have updated the terminology to reflect this more accurate description. The revision can be found on page 2, line 68-71. Thank you again for pointing this out, and we hope this modification improves the clarity and accuracy of the manuscript.

3. **Reviewer’ s comment: Page 3, Line 83: The following sentence is not clear. Please clarify: “However, the heterogeneity of disease manifestation among ARDS patients is ignored when only using lower tidal volume ventilation calculated by ideal body weight or limited platform pressure.” Do the authors mean that the current lung protective strategies do not take disease heterogeneity into account? And, what do the authors mean by disease heterogeneity? Severity, etiology or other?**

**Response:** We appreciate the reviewer’s observation and have reworded this sentence for clarity. Our intention was to convey that with the growing understanding of ARDS pathophysiology and advancements in target techniques, using only tidal volume and plateau pressure control as the sole methods for lung-protective ventilation may not be beneficial for all patients. Specifically, such strategies may not adequately address the heterogeneity of ARDS patients. By heterogeneity, we mean the differences in etiology, pathology, biological characteristics, and treatment responses among ARDS patients. These differences highlight that a one-size-fits-all approach may not always lead to optimal outcomes. We have revised this part of the manuscript to more clearly express these points. The updated statement can be found on page 2, line 71-74. The revised sentence now reads: *"However, current lung protective strategies, such as using lower tidal volume ventilation calculated by ideal body weight, fail to address the heterogeneity in disease manifestation among ARDS patients, which includes differences in disease severity and individual lung compliance[1-2].*"Thank you again for your valuable feedback, and we hope this revision provides the necessary clarification.

[1] Deans KJ, Minneci PC, Cui X, et al. Mechanical ventilation in ARDS: One size does not fit all[J]. Crit Care Med, 2005, 33(5):1141-1143.

[2] Goligher EC, Costa ELV, Yarnell CJ, et al. Effect of Lowering Vt on Mortality in Acute Respiratory Distress Syndrome Varies with Respiratory System Elastance[J]. Am J Respir Crit Care Med, 2021, 203(11):1378-1385.

4. **Reviewer’s comment: Page 3, Line 87--A recent meta-analysis conducted through a randomized control trial by the authors found that mechanical ventilation 88 guided by TPDP was associated with decreased mortality among patients with ARDS.**

**Response:** We apologize for our initial phrasing may have caused misunderstanding. The meta-analysis we referenced was published in 2023 and assessed the role and effects of mechanical ventilation guided by transpulmonary pressure (TPDP) in patients with ARDS. The meta-analysis included 13 randomized controlled trials (RCTs) that specifically investigated the use of TPP in ARDS patients. The revised manuscript now clearly reflects this, and the updated sentence can be found on page 3, line 77-80. The sentence has been revised to accurately reflect the methodology. It now reads: “*A recent meta-analysis of randomized controlled trials conducted by the authors found that mechanical ventilation guided by transpulmonary pressure was associated with decreased mortality among patients with ARDS*.” We sincerely apologize for the confusion caused by the previous wording and hope this revision resolves the issue.

5. **Reviewer’s comment: Page 3, Line 90--Please reword the following to clarify the concept: Pirrone’ study found that end-expiratory transpulmonary pressure above 0 cm 90 H2O promoted collapsed alveolar reopen**

**Response:** Thank you for the reviewer’s valuable comment. We apologize for the misunderstanding caused by our initial expression. We understand that our original phrasing was unclear and may have led to confusion. What we intended to convey is that Pirrone’s study found that during mechanical ventilation, target the end-expiratory transpulmonary pressure and adjusting parameters like PEEP to maintain the end-expiratory transpulmonary pressure above 0 cm H2O can help reopen collapsed alveoli, thereby improving lung compliance and oxygenation index. We sincerely apologize for the confusion caused by our previous wording, and we have revised the manuscript to clarify this point. The updated sentence can be found on page 3, line 80-82. The revised sentence now reads: ”*Pirrone's study found that an end-expiratory transpulmonary pressure above 0 cm H2O facilitated the reopening of collapsed alveoli*.”

6. **Reviewer’s comment: Page 3, Line 95--Please provide a reference for this statement: However, excessive transpulmonary pressure may lead to alveolar overdistension, raising the occurrence of VILI.**

**Response:** Thank you for the reviewer’s suggestion. We have added the following key references to this statement, covering the underlying mechanisms, animal studies, and clinical research, to strengthen the scientific basis. I have added the appropriate reference to support this statement: "*Excessive transpulmonary pressure may lead to alveolar overdistension, thereby increasing the risk of ventilator-induced lung injury (VILI)* [(1) Ball L, Talmor D, Pelosi P. Transpulmonary pressure target in critically ill patients: pros and cons. Crit Care. 2024 May 25;28(1):177. doi: 10.1186/s13054-024-04950-y; (2) Protti A, Andreis DT, Monti M, Santini A, Sparacino CC, Langer T, Votta E, Gatti S, Lombardi L, Leopardi O, Masson S, Cressoni M, Gattinoni L. Lung stress and strain during mechanical ventilation: any difference between statics and dynamics? Crit Care Med. 2013 Apr;41(4):1046-55. doi: 10.1097/CCM.0b013e31827417a6; (3) Sarge T, Loring SH, Yitsak-Sade M, Malhotra A, Novack V, Talmor D. Raising positive end-expiratory pressures in ARDS to achieve a positive transpulmonary pressure does not cause hemodynamic compromise. Intensive Care Med. 2014 Jan;40(1):126-8. doi: 10.1007/s00134-013-3127-1. Epub 2013 Oct 15. PMID: 24126673. ]."

7. **Reviewer’ s comment: Can the authors please include the tests used to determine distribution of the data (e.g., normal vs skewed)?**

**Response:** Thank you for the reviewer’s valuable feedback and for highlighting the need to include tests for data distribution. In response to the reviewer’s suggestion, we have now added a description of the tests used to assess the distribution of the data. Specifically, we used the Shapiro-Wilk test to test for normality. If the data followed a normal distribution, we used parametric tests. For non-normally distributed data, we used non-parametric tests. The updated sentence can be found on page 5, line 157-162.

Revised Text in the Statistical Analysis Section:

*Descriptive Statistics*

*Normality was assessed using the Shapiro-Wilk test. Continuous variables following a normal distribution are expressed as mean ± standard deviation (MD ± SD), with comparisons between two groups conducted using the t-test. Non-normally distributed continuous variables are presented as median (interquartile range) [Median (IQR)], with comparisons performed using the Wilcoxon test. Categorical variables are expressed as percentages.*

**Table 2 Normality Test of variables in TPDP Group and No-TPDP Group.**

| Variable | Test_Scope | W_statistic | P_value | N |
| --- | --- | --- | --- | --- |
| Age | All | 0.984 | 0.000 | 4721 |
| Age | No-TPDP group | 0.983 | 0.000 | 4426 |
| Age | TPDP group | 0.984 | 0.002 | 295 |
| BMI | All | 0.899 | 0.000 | 4721 |
| BMI | No-TPDP group | 0.902 | 0.000 | 4426 |
| BMI | TPDP group | 0.907 | 0.000 | 295 |
| SOFA_score | All | 0.968 | 0.000 | 4721 |
| SOFA_score | No-TPDP group | 0.965 | 0.000 | 4426 |
| SOFA_score | TPDP group | 0.988 | 0.012 | 295 |
| APSIII | All | 0.980 | 0.000 | 4721 |
| APSIII | No-TPDP group | 0.980 | 0.000 | 4426 |
| APSIII | TPDP group | 0.990 | 0.047 | 295 |
| ABPd | All | 0.983 | 0.000 | 4721 |
| ABPd | No-TPDP group | 0.982 | 0.000 | 4426 |
| ABPd | TPDP group | 0.987 | 0.008 | 295 |
| ABPs | All | 0.962 | 0.000 | 4721 |
| ABPs | No-TPDP group | 0.962 | 0.000 | 4426 |
| ABPs | TPDP group | 0.967 | 0.000 | 295 |
| Heart_Rate | All | 0.993 | 0.000 | 4721 |
| Heart_Rate | No-TPDP group | 0.993 | 0.000 | 4426 |
| Heart_Rate | TPDP group | 0.992 | 0.088 | 295 |
| WBC | All | 0.990 | 0.000 | 4721 |
| WBC | No-TPDP group | 0.990 | 0.000 | 4426 |
| WBC | TPDP group | 0.988 | 0.019 | 295 |
| Creatinine | All | 0.633 | 0.000 | 4721 |
| Creatinine | No-TPDP group | 0.639 | 0.000 | 4426 |
| Creatinine | TPDP group | 0.564 | 0.000 | 295 |
| Glucose | All | 0.985 | 0.000 | 4721 |
| Glucose | No-TPDP group | 0.985 | 0.000 | 4426 |
| Glucose | TPDP group | 0.982 | 0.001 | 295 |
| BUN | All | 0.900 | 0.000 | 4721 |
| BUN | No-TPDP group | 0.899 | 0.000 | 4426 |
| BUN | TPDP group | 0.916 | 0.000 | 295 |
| PT | All | 0.920 | 0.000 | 4721 |
| PT | No-TPDP group | 0.918 | 0.000 | 4426 |
| PT | TPDP group | 0.949 | 0.000 | 295 |
| PlateletCount | All | 0.967 | 0.000 | 4721 |
| PlateletCount | No-TPDP group | 0.967 | 0.000 | 4426 |
| PlateletCount | TPDP group | 0.967 | 0.000 | 295 |
| Lac | All | 0.939 | 0.000 | 4721 |
| Lac | No-TPDP group | 0.940 | 0.000 | 4426 |
| Lac | TPDP group | 0.936 | 0.000 | 295 |
| Arterial_PH | All | 0.854 | 0.000 | 4721 |
| Arterial_PH | No-TPDP group | 0.850 | 0.000 | 4426 |
| Arterial_PH | TPDP group | 0.836 | 0.000 | 295 |
| Arterial_O2_pressure | All | 0.970 | 0.000 | 4721 |
| Arterial_O2_pressure | No-TPDP group | 0.971 | 0.000 | 4426 |
| Arterial_O2_pressure | TPDP group | 0.951 | 0.000 | 295 |
| Arterial_CO2_Pressure | All | 0.993 | 0.000 | 4721 |
| Arterial_CO2_Pressure | No-TPDP group | 0.993 | 0.000 | 4426 |
| Arterial_CO2_Pressure | TPDP group | 0.990 | 0.049 | 295 |
| HCO3 | All | 0.992 | 0.000 | 4721 |
| HCO3 | No-TPDP group | 0.992 | 0.000 | 4426 |
| HCO3 | TPDP group | 0.988 | 0.017 | 295 |
| PFratio | All | 0.960 | 0.000 | 4721 |
| PFratio | No-TPDP group | 0.961 | 0.000 | 4426 |
| PFratio | TPDP group | 0.899 | 0.000 | 295 |
| Total_Respiratory_Rate | All | 0.984 | 0.000 | 4721 |
| Total_Respiratory_Rate | No-TPDP group | 0.984 | 0.000 | 4426 |
| Total_Respiratory_Rate | TPDP group | 0.978 | 0.000 | 295 |
| Spontaneous_Respiratory_Rate | All | 0.702 | 0.000 | 4721 |
| Spontaneous_Respiratory_Rate | No-TPDP group | 0.715 | 0.000 | 4426 |
| Spontaneous_Respiratory_Rate | TPDP group | 0.427 | 0.000 | 295 |
| Set_Respiratory_Rate | All | 0.972 | 0.000 | 4721 |
| Set_Respiratory_Rate | No-TPDP group | 0.972 | 0.000 | 4426 |
| Set_Respiratory_Rate | TPDP group | 0.966 | 0.000 | 295 |
| Tidal_Volume | All | 1.000 | 0.671 | 4721 |
| Tidal_Volume | No-TPDP group | 1.000 | 0.746 | 4426 |
| Tidal_Volume | TPDP group | 0.996 | 0.571 | 295 |
| PEEP | All | 0.828 | 0.000 | 4721 |
| PEEP | No-TPDP group | 0.826 | 0.000 | 4426 |
| PEEP | TPDP group | 0.889 | 0.000 | 295 |
| Plateau_Pressure | All | 0.984 | 0.000 | 4721 |
| Plateau_Pressure | No-TPDP group | 0.987 | 0.000 | 4426 |
| Plateau_Pressure | TPDP group | 0.987 | 0.009 | 295 |
| Peak_Pressure | All | 0.992 | 0.000 | 4721 |
| Peak_Pressure | No-TPDP group | 0.993 | 0.000 | 4426 |
| Peak_Pressure | TPDP group | 0.991 | 0.069 | 295 |
| Lung_compliance | All | 0.722 | 0.000 | 4721 |
| Lung_compliance | No-TPDP group | 0.710 | 0.000 | 4426 |
| Lung_compliance | TPDP group | 0.881 | 0.000 | 295 |
| Driving_pressure | All | 0.983 | 0.000 | 4721 |
| Driving_pressure | No-TPDP group | 0.984 | 0.000 | 4426 |
| Driving_pressure | TPDP group | 0.993 | 0.178 | 295 |
| Mechanical_power | All | 0.943 | 0.000 | 4721 |
| Mechanical_power | No-TPDP group | 0.947 | 0.000 | 4426 |
| Mechanical_power | TPDP group | 0.962 | 0.000 | 295 |
| Mechanical_ventilation_hour | All | 0.701 | 0.000 | 4721 |
| Mechanical_ventilation_hour | No-TPDP group | 0.689 | 0.000 | 4426 |
| Mechanical_ventilation_hour | TPDP group | 0.866 | 0.000 | 295 |

**Table 3 Normality Test of variables in TPDP Group and No-TPDP Group in matched cohort.**

| Variable | Test_Scope | W_statistic | P_value | N |
| --- | --- | --- | --- | --- |
| Age | All | 0.986 | 0.000 | 526 |
| Age | No-TPDP group | 0.987 | 0.014 | 263 |
| Age | TPDP group | 0.982 | 0.002 | 263 |
| BMI | All | 0.940 | 0.000 | 526 |
| BMI | No-TPDP group | 0.936 | 0.000 | 263 |
| BMI | TPDP group | 0.937 | 0.000 | 263 |
| SOFA_score | All | 0.986 | 0.000 | 526 |
| SOFA_score | No-TPDP group | 0.981 | 0.001 | 263 |
| SOFA_score | TPDP group | 0.984 | 0.004 | 263 |
| APSIII | All | 0.988 | 0.000 | 526 |
| APSIII | No-TPDP group | 0.982 | 0.002 | 263 |
| APSIII | TPDP group | 0.990 | 0.074 | 263 |
| ABPd | All | 0.985 | 0.000 | 526 |
| ABPd | No-TPDP group | 0.980 | 0.001 | 263 |
| ABPd | TPDP group | 0.987 | 0.021 | 263 |
| ABPs | All | 0.978 | 0.000 | 526 |
| ABPs | No-TPDP group | 0.984 | 0.005 | 263 |
| ABPs | TPDP group | 0.968 | 0.000 | 263 |
| Heart_Rate | All | 0.990 | 0.001 | 526 |
| Heart_Rate | No-TPDP group | 0.979 | 0.001 | 263 |
| Heart_Rate | TPDP group | 0.992 | 0.197 | 263 |
| WBC | All | 0.990 | 0.001 | 526 |
| WBC | No-TPDP group | 0.988 | 0.027 | 263 |
| WBC | TPDP group | 0.985 | 0.007 | 263 |
| Creatinine | All | 0.617 | 0.000 | 526 |
| Creatinine | No-TPDP group | 0.767 | 0.000 | 263 |
| Creatinine | TPDP group | 0.530 | 0.000 | 263 |
| Glucose | All | 0.981 | 0.000 | 526 |
| Glucose | No-TPDP group | 0.974 | 0.000 | 263 |
| Glucose | TPDP group | 0.982 | 0.002 | 263 |
| BUN | All | 0.916 | 0.000 | 526 |
| BUN | No-TPDP group | 0.918 | 0.000 | 263 |
| BUN | TPDP group | 0.912 | 0.000 | 263 |
| PT | All | 0.959 | 0.000 | 526 |
| PT | No-TPDP group | 0.962 | 0.000 | 263 |
| PT | TPDP group | 0.956 | 0.000 | 263 |
| PlateletCount | All | 0.966 | 0.000 | 526 |
| PlateletCount | No-TPDP group | 0.965 | 0.000 | 263 |
| PlateletCount | TPDP group | 0.963 | 0.000 | 263 |
| Lac | All | 0.949 | 0.000 | 526 |
| Lac | No-TPDP group | 0.955 | 0.000 | 263 |
| Lac | TPDP group | 0.934 | 0.000 | 263 |
| Arterial_PH | All | 0.847 | 0.000 | 526 |
| Arterial_PH | No-TPDP group | 0.858 | 0.000 | 263 |
| Arterial_PH | TPDP group | 0.833 | 0.000 | 263 |
| Arterial_O2_pressure | All | 0.952 | 0.000 | 526 |
| Arterial_O2_pressure | No-TPDP group | 0.952 | 0.000 | 263 |
| Arterial_O2_pressure | TPDP group | 0.947 | 0.000 | 263 |
| Arterial_CO2_Pressure | All | 0.993 | 0.010 | 526 |
| Arterial_CO2_Pressure | No-TPDP group | 0.992 | 0.138 | 263 |
| Arterial_CO2_Pressure | TPDP group | 0.991 | 0.096 | 263 |
| HCO3 | All | 0.986 | 0.000 | 526 |
| HCO3 | No-TPDP group | 0.984 | 0.005 | 263 |
| HCO3 | TPDP group | 0.986 | 0.010 | 263 |
| PFratio | All | 0.908 | 0.000 | 526 |
| PFratio | No-TPDP group | 0.901 | 0.000 | 263 |
| PFratio | TPDP group | 0.911 | 0.000 | 263 |
| Total_Respiratory_Rate | All | 0.986 | 0.000 | 526 |
| Total_Respiratory_Rate | No-TPDP group | 0.987 | 0.017 | 263 |
| Total_Respiratory_Rate | TPDP group | 0.981 | 0.001 | 263 |
| Spontaneous_Respiratory_Rate | All | 0.467 | 0.000 | 526 |
| Spontaneous_Respiratory_Rate | No-TPDP group | 0.480 | 0.000 | 263 |
| Spontaneous_Respiratory_Rate | TPDP group | 0.449 | 0.000 | 263 |
| Set_Respiratory_Rate | All | 0.978 | 0.000 | 526 |
| Set_Respiratory_Rate | No-TPDP group | 0.977 | 0.000 | 263 |
| Set_Respiratory_Rate | TPDP group | 0.974 | 0.000 | 263 |
| Tidal_Volume | All | 0.998 | 0.796 | 526 |
| Tidal_Volume | No-TPDP group | 0.988 | 0.024 | 263 |
| Tidal_Volume | TPDP group | 0.994 | 0.361 | 263 |
| PEEP | All | 0.936 | 0.000 | 526 |
| PEEP | No-TPDP group | 0.936 | 0.000 | 263 |
| PEEP | TPDP group | 0.937 | 0.000 | 263 |
| Plateau_Pressure | All | 0.992 | 0.009 | 526 |
| Plateau_Pressure | No-TPDP group | 0.989 | 0.046 | 263 |
| Plateau_Pressure | TPDP group | 0.991 | 0.106 | 263 |
| Peak_Pressure | All | 0.992 | 0.009 | 526 |
| Peak_Pressure | No-TPDP group | 0.986 | 0.010 | 263 |
| Peak_Pressure | TPDP group | 0.995 | 0.526 | 263 |
| Lung_compliance | All | 0.891 | 0.000 | 526 |
| Lung_compliance | No-TPDP group | 0.887 | 0.000 | 263 |
| Lung_compliance | TPDP group | 0.888 | 0.000 | 263 |
| Driving_pressure | All | 0.995 | 0.084 | 526 |
| Driving_pressure | No-TPDP group | 0.990 | 0.082 | 263 |
| Driving_pressure | TPDP group | 0.993 | 0.265 | 263 |
| Mechanical_power | All | 0.968 | 0.000 | 526 |
| Mechanical_power | No-TPDP group | 0.965 | 0.000 | 263 |
| Mechanical_power | TPDP group | 0.967 | 0.000 | 263 |
| Mechanical_ventilation_hour | All | 0.815 | 0.000 | 526 |
| Mechanical_ventilation_hour | No-TPDP group | 0.765 | 0.000 | 263 |
| Mechanical_ventilation_hour | TPDP group | 0.848 | 0.000 | 263 |

**Table 4 Normality Test of variables in TPDP≤12.5cmH2O group and TPDP＞12.5cmH2O group.**

| Variable | Test_Scope | W_statistic | P_value | N |
| --- | --- | --- | --- | --- |
| Age | All | 0.984 | 0.002 | 295 |
| Age | TPDP≤12.5cmH2O group | 0.981 | 0.003 | 231 |
| Age | TPDP＞12.5cmH2O group | 0.99 | 0.896 | 64 |
| BMI | All | 0.907 | 0.000 | 295 |
| BMI | TPDP≤12.5cmH2O group | 0.886 | 0.000 | 231 |
| BMI | TPDP＞12.5cmH2O group | 0.955 | 0.020 | 64 |
| SOFA_score | All | 0.988 | 0.012 | 295 |
| SOFA_score | TPDP≤12.5cmH2O group | 0.989 | 0.081 | 231 |
| SOFA_score | TPDP＞12.5cmH2O group | 0.972 | 0.150 | 64 |
| APSIII | All | 0.99 | 0.047 | 295 |
| APSIII | TPDP≤12.5cmH2O group | 0.991 | 0.172 | 231 |
| APSIII | TPDP＞12.5cmH2O group | 0.971 | 0.144 | 64 |
| ABPd | All | 0.987 | 0.008 | 295 |
| ABPd | TPDP≤12.5cmH2O group | 0.98 | 0.003 | 231 |
| ABPd | TPDP＞12.5cmH2O group | 0.984 | 0.565 | 64 |
| ABPs | All | 0.967 | 0.000 | 295 |
| ABPs | TPDP≤12.5cmH2O group | 0.962 | 0.000 | 231 |
| ABPs | TPDP＞12.5cmH2O group | 0.968 | 0.091 | 64 |
| Heart_Rate | All | 0.992 | 0.088 | 295 |
| Heart_Rate | TPDP≤12.5cmH2O group | 0.987 | 0.040 | 231 |
| Heart_Rate | TPDP＞12.5cmH2O group | 0.988 | 0.784 | 64 |
| WBC | All | 0.988 | 0.019 | 295 |
| WBC | TPDP≤12.5cmH2O group | 0.986 | 0.027 | 231 |
| WBC | TPDP＞12.5cmH2O group | 0.986 | 0.673 | 64 |
| Creatinine | All | 0.564 | 0.000 | 295 |
| Creatinine | TPDP≤12.5cmH2O group | 0.519 | 0.000 | 231 |
| Creatinine | TPDP＞12.5cmH2O group | 0.828 | 0.000 | 64 |
| Glucose | All | 0.982 | 0.001 | 295 |
| Glucose | TPDP≤12.5cmH2O group | 0.981 | 0.004 | 231 |
| Glucose | TPDP＞12.5cmH2O group | 0.972 | 0.159 | 64 |
| BUN | All | 0.916 | 0.000 | 295 |
| BUN | TPDP≤12.5cmH2O group | 0.906 | 0.000 | 231 |
| BUN | TPDP＞12.5cmH2O group | 0.927 | 0.001 | 64 |
| PT | All | 0.949 | 0.000 | 295 |
| PT | TPDP≤12.5cmH2O group | 0.945 | 0.000 | 231 |
| PT | TPDP＞12.5cmH2O group | 0.954 | 0.018 | 64 |
| PlateletCount | All | 0.967 | 0.000 | 295 |
| PlateletCount | TPDP≤12.5cmH2O group | 0.965 | 0.000 | 231 |
| PlateletCount | TPDP＞12.5cmH2O group | 0.97 | 0.125 | 64 |
| Lac | All | 0.936 | 0.000 | 295 |
| Lac | TPDP≤12.5cmH2O group | 0.941 | 0.000 | 231 |
| Lac | TPDP＞12.5cmH2O group | 0.91 | 0.000 | 64 |
| Arterial_PH | All | 0.836 | 0.000 | 295 |
| Arterial_PH | TPDP≤12.5cmH2O group | 0.832 | 0.000 | 231 |
| Arterial_PH | TPDP＞12.5cmH2O group | 0.83 | 0.000 | 64 |
| Arterial_O2_pressure | All | 0.951 | 0.000 | 295 |
| Arterial_O2_pressure | TPDP≤12.5cmH2O group | 0.95 | 0.000 | 231 |
| Arterial_O2_pressure | TPDP＞12.5cmH2O group | 0.935 | 0.002 | 64 |
| Arterial_CO2_Pressure | All | 0.99 | 0.049 | 295 |
| Arterial_CO2_Pressure | TPDP≤12.5cmH2O group | 0.993 | 0.388 | 231 |
| Arterial_CO2_Pressure | TPDP＞12.5cmH2O group | 0.966 | 0.078 | 64 |
| HCO3 | All | 0.988 | 0.017 | 295 |
| HCO3 | TPDP≤12.5cmH2O group | 0.987 | 0.029 | 231 |
| HCO3 | TPDP＞12.5cmH2O group | 0.986 | 0.661 | 64 |
| PFratio | All | 0.899 | 0.000 | 295 |
| PFratio | TPDP≤12.5cmH2O group | 0.899 | 0.000 | 231 |
| PFratio | TPDP＞12.5cmH2O group | 0.89 | 0.000 | 64 |
| Total_Respiratory_Rate | All | 0.978 | 0.000 | 295 |
| Total_Respiratory_Rate | TPDP≤12.5cmH2O group | 0.978 | 0.001 | 231 |
| Total_Respiratory_Rate | TPDP＞12.5cmH2O group | 0.958 | 0.029 | 64 |
| Spontaneous_Respiratory_Rate | All | 0.427 | 0.000 | 295 |
| Spontaneous_Respiratory_Rate | TPDP≤12.5cmH2O group | 0.453 | 0.000 | 231 |
| Spontaneous_Respiratory_Rate | TPDP＞12.5cmH2O group | 0.364 | 0.000 | 64 |
| Set_Respiratory_Rate | All | 0.966 | 0.000 | 295 |
| Set_Respiratory_Rate | TPDP≤12.5cmH2O group | 0.967 | 0.000 | 231 |
| Set_Respiratory_Rate | TPDP＞12.5cmH2O group | 0.956 | 0.024 | 64 |
| Tidal_Volume | All | 0.996 | 0.571 | 295 |
| Tidal_Volume | TPDP≤12.5cmH2O group | 0.991 | 0.195 | 231 |
| Tidal_Volume | TPDP＞12.5cmH2O group | 0.987 | 0.753 | 64 |
| PEEP | All | 0.889 | 0.000 | 295 |
| PEEP | TPDP≤12.5cmH2O group | 0.903 | 0.000 | 231 |
| PEEP | TPDP＞12.5cmH2O group | 0.862 | 0.000 | 64 |
| Plateau_Pressure | All | 0.987 | 0.009 | 295 |
| Plateau_Pressure | TPDP≤12.5cmH2O group | 0.988 | 0.050 | 231 |
| Plateau_Pressure | TPDP＞12.5cmH2O group | 0.952 | 0.014 | 64 |
| Peak_Pressure | All | 0.991 | 0.069 | 295 |
| Peak_Pressure | TPDP≤12.5cmH2O group | 0.993 | 0.302 | 231 |
| Peak_Pressure | TPDP＞12.5cmH2O group | 0.97 | 0.122 | 64 |
| Lung_compliance | All | 0.881 | 0.000 | 295 |
| Lung_compliance | TPDP≤12.5cmH2O group | 0.874 | 0.000 | 231 |
| Lung_compliance | TPDP＞12.5cmH2O group | 0.92 | 0.000 | 64 |
| Driving_pressure | All | 0.993 | 0.178 | 295 |
| Driving_pressure | TPDP≤12.5cmH2O group | 0.989 | 0.073 | 231 |
| Driving_pressure | TPDP＞12.5cmH2O group | 0.992 | 0.941 | 64 |
| Mechanical_power | All | 0.962 | 0.000 | 295 |
| Mechanical_power | TPDP≤12.5cmH2O group | 0.958 | 0.000 | 231 |
| Mechanical_power | TPDP＞12.5cmH2O group | 0.97 | 0.116 | 64 |
| Mechanical_ventilation_hour | All | 0.866 | 0.000 | 295 |
| Mechanical_ventilation_hour | TPDP≤12.5cmH2O group | 0.859 | 0.000 | 231 |
| Mechanical_ventilation_hour | TPDP＞12.5cmH2O group | 0.893 | 0.000 | 64 |

8. **Reviewer’ s comment: Can the authors please provide more information about “spontaneous respiration” under propensity matching variables? Is this over breathing the set respiratory rate or something else?**

**Response:** “Spontaneous respiration" refers to the patient’s ability to breathe independently, without mechanical assistance.It refers to the spontaneous breaths taken by the patient in addition to the set ventilator rate, which could indicate the patient's respiratory effort or level of respiratory function. This variable was included in the propensity matching analysis to reduce the confounding bias of spontaneous breathing on the clinical outcomes.

9. **Reviewer’ s comment: The Discussion still needs work to have a clear presentation.**

**The Discussion is a literary forum that is used to interpret your findings and put your data in context of the existing literature. This Discussion reads more like a review of the literature rather than a scientific discussion of the study.**

**Response:** Thank you for your constructive feedback and the opportunity to improve our manuscript. We have carefully addressed all points raised in your review, with revisions focused on strengthening the Discussion section’s clarity, scientific rigor, and clinical relevance. Below is a point-by-point response to your comments:

1) **start your Discussion with the paragraph starting at line 412 on page 13.**

As suggested, we restructured the Discussion to begin with the paragraph on line 412 (Page 13) of the original manuscript. This section now opens with our primary findings on the association between elevated transpulmonary driving pressure (TPDP) and mortality, mediated by peak airway pressure. We explicitly framed these results within the context of ventilator-induced lung injury mechanisms and phenotype-specific responses.

Revised Text:

*Phenotype-Specific Mechanisms and Clinical Implications of TPDP in ARDS*

*Our study demonstrates that elevated transpulmonary driving pressure shows significant association with increased mortality in acute respiratory distress syndrome patients. Recent investigations have further underscored the critical prognostic implications the TPDP in ARDS management. Placenti et al. [22] established that TPDP more reliably reflects lung distension pressures compared to airway driving pressure, representing the actual transpulmonary pressure gradient without the distending pressure generated by the chest wall or esophageal driving pressure. In a study by Baedorf Kassis et al [23], treatment strategies leading to decreased transpulmonary driving pressure may be associated with improved 28 day mortality. This finding is consistent with the 2024 study by Sun et al, where TPDP should be used as a key target indicator for lung protective ventilation [24].*

*Our study further revealed that when TPDP exceeds 12.5 cmH₂O, patient mortality significantly increases. This threshold closely aligns with the 'safety window' (10–12 cmH₂O) proposed by Placenti et al. [22], suggesting that 12.5 cmH₂O may represent the critical threshold for triggering ventilator-induced lung injury (VILI). This relationship may be partially mediated through higher peak airway pressure. The detrimental clinical impacts of elevated peak airway pressures have been well documented. Experimental evidence from a rat mechanical ventilation model developed by Katira et al. [25] revealed that excessive peak airway pressure induce right ventricular overload through increased pulmonary vascular capacitance and perfusion abnormalities. It is suggested that control of peak airway pressure can interrupt the vicious cycle of "high TPDP→ alveolar hyperinflation → pulmonary circulation injury". Our causal mediation analysis substantiates the hypothesis that therapeutic strategies targeting TPDP reduction could mitigate both peak airway pressure elevation and subsequent pulmonary damage. This mechanistic understanding emphasizes the potential clinical utility of TPDP-guided ventilation strategies in ARDS management.*

*The differential effects of transpulmonary driving pressure (TPDP) across ARDS phenotypes arise from distinct pathophysiological mechanisms and heterogeneous lung mechanical properties. In Phenotype I (High Mechanical Power with Moderate Lung Compliance), the preserved lung compliance indicates partial alveolar recruitability. However, excessive mechanical power potentiates VILI via bioenergetic stress-induced tissue damage and inflammatory cascades[26], where elevated TPDP potentiates ventilator-induced lung injury through biomechanical transduction pathways. Phenotype II (High Spontaneous Effort with Elevated Compliance) demonstrates paradoxically increased lung compliance combined with vigorous spontaneous breathing effort, creating conditions favorable for pendelluft phenomenon[27]. In this context, TPDP elevation amplifies cyclic alveolar collapse-reopening cycles in dependent lung zones, thereby exacerbating atelectrauma through shear stress mechanisms[28]. Phenotype III (Low Tidal Volume with Reduced Compliance), characterized by low tidal volume and markedly diminished compliance, exhibits limited TPDP transmission due to mechanical stress dissipation through non-recruitable lung parenchyma. This stress-shielding effect explains the absence of significant mortality correlation despite TPDP fluctuations. This mechanistic stratification underscores the clinical imperative for phenotype-driven ventilation strategies. Our observations harmonize with contemporary evidence from Liu et al. [29], reinforcing the paradigm shift toward precision medicine in ARDS management.*

The updated statement can be found on page 11-12, line 342-390.

2) **Then please discuss the pros and cons of your data source: using a database for your analysis as opposed to performing a clinical trial. Consider the reliability of the databank and the type of information. While this database was rich in patient variables, it was not designed to support an analysis of outcomes for etiology. Therefore, it lacks details that are needed for direct correlation analysis. This explains why your study lacks information on causation and why you needed a substitute mediation analysis. However, as pointed out a medication analysis is still statistical conjecture that requires further testing. Further, it lacks practical description needed by clinicians for patient application.**

We added a subsection titled “Strengths and Limitations of Database Utilization” to address the pros and cons of using MIMIC-IV. This includes: (1) Pros: Large-scale data, multi-institutional generalizability, detailed respiratory parameters. (2) Cons: Lack of granular data on ARDS etiology, therapeutic interventions, and bedside-level details. (3) Statistical Limitations: Mediation analysis as a substitute for direct causation and its inferential nature.

Revised Text:

***Strengths and Limitations of Database Utilization***

*MIMIC-IV enables large-scale retrospective ARDS analysis, enhancing generalizability. Its detailed respiratory and physiological data facilitate an in-depth evaluation of TPDP. However, it lacks granular details on ARDS onset, therapeutic interventions, and individualized management, precluding direct causality assessment. Mediation analysis provides inferential insights but requires validation in prospective studies. Additionally, the absence of bedside-level details limits direct clinical applicability.*

The updated statement can be found on page 13, line 404-411.

3) **Discuss the internal and external validity of your findings based on the methods you used. This will require a critical appraisal of your statistical methods. You can point out that your data source is from multiple institutions and therefore can be generalized to other practice sites with similar resources and facilities to those that participated in the data base. It should be noted that these findings may not be applicable to clinical practice centers that differ from those in the data source.**

A new subsection “Internal and External Validity Considerations” was added. We critically appraised our statistical methods (propensity score matching, mediation analysis) and clarified generalizability boundaries: Internal Validity: Addressed residual confounding risks. External Validity: Highlighted applicability to centers with similar resources, while cautioning against those differ from the data source.

Revised Text:

***Internal and External Validity Considerations***

*The study’s internal validity is reinforced by rigorous statistical adjustments, including propensity score matching and mediation analysis, aimed at reducing confounding effects. However, given the retrospective design, residual confounding cannot be entirely ruled out due to potential unmeasured variables. External validity is supported by the multi-institutional nature of the MIMIC-IV database, allowing broader applicability to healthcare centers with similar resources and clinical practices. Nonetheless, findings may not be generalizable to institutions with different patient populations, ventilatory management protocols, or resource availability, highlighting the need for validation in diverse clinical environments.*

The updated statement can be found on page 13, line 412-421.

4) **Summarize the overall limitations of this study and what has to be done to overcome the issues. (For example, you could say that while statistical analysis of the database provided preliminary evidence that careful control of TPDP could improve ARDS outcomes, controlled clinical trials are still necessary to identify all confounders in outcomes and the effects of various interventions taken to achieve a TPDP of 12.5 cm or less.**

We expanded the “Study Limitations and Future Directions” section to explicitly align with the recommendations: Key Limitations: Observational design, lack of standardized TPDP protocols. Future Work: Emphasis on prospective trials to validate TPDP thresholds and phenotype-specific strategies.

Revised Text:

***Study Limitations and Future Directions***

*Several limitations should be considered when interpreting our findings. First, the observational study design prevents definitive causal inference. Although our statistical models suggest an association between TPDP and mortality, prospective randomized trials are required to confirm these findings. Second, the lack of standardized TPDP measurement protocols within MIMIC-IV introduces potential variability in data accuracy and reproducibility. Third, while our phenotype-based subgroup analysis provides novel insights into differential responses to TPDP, further validation through prospective cohort studies and mechanistic investigations is needed. Future research should prioritize controlled clinical trials to determine the optimal TPDP threshold, clarify its interaction with mechanical power, and evaluate the impact of phenotype-specific ventilatory strategies on ARDS outcomes.*

The updated statement can be found on page 13, line 423-433.

5) **Provide precise conclusions based on the entirety of the Discussion.**

The Conclusions section was streamlined to focus on actionable insights derived from the Discussion: Prioritized TPDP-guided ventilation for mechanical stress-dominant phenotypes. Highlighted the need for controlled trials to confirm thresholds.

Revised Text:

*Our study suggests that TPDP >12.5 cmH2O may be associated with an increased risk of mortality in ARDS patients, particularly those with moderate to severe ARDS. Incorporating target TPDP values into routine clinical practice might enhance the precision of mechanical ventilation strategies, potentially reducing ventilator-induced lung injury and improving patient outcomes. However, recognizing the different ARDS phenotypes and tailoring ventilation strategies based on these profiles could further contribute to better patient prognosis. Future research should aim to validate these findings, develop phenotype-specific management protocols, and explore the mechanisms underlying the varied responses to TPDP in ARDS patients. This will help provide a more comprehensive understanding of the role of TPDP in the management of ARDS.*

The updated statement can be found on page 13-14, line 435-445.

**6) Please remove all descriptive information in the Discussion unless it is needed to support a statement in the 5 major sections described above. Right now, your Discussion is closer to a review and fails to interpret your data.**

All non-essential descriptive text (e.g., background reviews of Berlin criteria) was removed. The Discussion now strictly interprets data through the lens of your requested themes. These revisions significantly enhance the manuscript’s scientific value. Thank you again for your time and expertise. Please let us know if further adjustments are needed.

**Responses to the Reviewer 2’s comments:**

1. **Reviewer’ s comment: Grammar and Spelling.**

**Response:** I sincerely apologize for the language issues in the manuscript. To address the reviewer’s concern, I have submitted the revised manuscript to a professional proofreading service to ensure that the grammar, spelling, and sentence structure are polished. The final version has undergone comprehensive language editing.

2. **Reviewer’ s comment: Abstract to be restructured and recreated in relation to comments below. I recommend in the abstract to eliminate all abbreviations which are never recommended in this part of the work as they only weigh down the work and the section, and make it complicated to read.**

**Response:** I have carefully revised the abstract as per the reviewer’s suggestion to eliminate abbreviations. The revised version is now free from abbreviations, making the abstract simpler and more accessible to readers. I have also ensured that the language is clear and concise to improve the overall readability of the section.

Revised Text in the Abstract Section:

*Background*

*This study examined the effect of target transpulmonary driving pressure on mortality in patients with Acute Respiratory Distress Syndrome, assessing how varying levels of transpulmonary driving pressure influence clinical outcomes.*

*Methods*

*This retrospective study utilized data from the MIMIC-IV database to evaluate the relationship between transpulmonary driving pressure and mortality in Acute Respiratory Distress Syndrome. Associations between transpulmonary driving pressure levels and 28-day, ICU, and hospital mortality were analyzed. Propensity score matching was employed to balance covariates, while causal mediation analysis assessed whether peak airway pressure mediated the effect of transpulmonary driving pressure on mortality.*

*Results*

*Among 4721 patients with Acute Respiratory Distress Syndrome, 295 received transpulmonary driving pressure targeting. The optimal transpulmonary driving pressure threshold was identified as 12.5 cmH2O. Patients with transpulmonary driving pressure >12.5 cmH2O had significantly higher 28-day, ICU, and hospital mortality, particularly in those with moderate to severe Acute Respiratory Distress Syndrome (p < 0.05). After propensity score matching, targeting transpulmonary driving pressure was associated with lower ICU mortality (HR 0.676, 95% CI 0.511–0.894, p = 0.006). Phenotypic analysis showed that elevated transpulmonary driving pressure was linked to worse outcomes in Phenotype-I(High Mechanical Power with Moderate Lung Compliance) and Phenotype-II (High Spontaneous Breathing with Better Lung Compliance), but not in Phenotype-III (Low Tidal Volume with Reduced Lung Compliance). Mediation analysis revealed that 7.0% of the mortality risk associated with transpulmonary driving pressure >12.5 cmH2O was mediated through peak airway pressure.*

*Conclusion*

*Transpulmonary driving pressure exceeding 12.5 cmH2O is associated with higher mortality in Acute Respiratory Distress Syndrome patients, with peak airway pressure contributing to this effect.*

The updated statement can be found on page 1-2, line 30-60.

3. **Reviewer’ s comment: I find the introduction adequate and well developed and argued. Some points to be clarified. Especially by including appropriate references to the statements made by the authors. Line 92-96 to insert a reference to this statement. Line 99-103 Insert an appropriate reference here as well.**

**Response:** I completely agree with this suggestion and have added the relevant references to support the statements made in these sections. This should help further clarify the content and provide stronger academic backing for the claims presented.

4. **Reviewer’ s comment: Materials and methods--Selection of patients: I do not understand why it was chosen by the authors to use the Berlin definition and not the new broader definition for ARDS criteria ( doi: 10.1164/rccm.202303-0558WS. ). To justify this choice.**

**Response:** Thank you for the reviewer’s insightful comment regarding our choice of the Berlin definition instead of the 2023 Global ARDS Definition. We appreciate the opportunity to clarify our rationale. We selected the Berlin definition for this study due to its strong compatibility with mechanical ventilation parameters, its well-validated prognostic stratification, and its feasibility for retrospective database research. Below, we provide a detailed justification:

1. Strong Compatibility with Mechanical Ventilation Parameters

The Berlin definition explicitly requires that PaO2/FiO2(P/F) ratio be assessed under PEEP ≥5 cmH2O, which allows for standardized ARDS severity classification (e.g., moderate ARDS:PaO2/FiO2≤200 mmHg). This criterion aligns closely with our study’s key respiratory mechanics variables, including transpulmonary driving pressure (TPDP), peak airway pressure, and mechanical power, ensuring consistency in mechanical ventilation settings across patients.

Ensuring parameter homogeneity: All patients in our study were mechanically ventilated with PEEP ≥5 cmH2O, preventing confounding effects from non-intubated patients included in the new definition (e.g., high-flow nasal oxygen therapy [HFNO ≥30 L/min]). This helps ensure a purer analysis of the causal relationship between TPDP and airway pressures.

2. Limitations of the 2023 Global ARDS Definition in Mechanically Ventilated Patients

While the 2023 Global ARDS Definition expands ARDS diagnostic criteria (e.g., including non-intubated patients and allowing SpO2/FiO2 as a substitute for PaO2/FiO2), its applicability to mechanically ventilated patients remains uncertain: Lack of standardized mechanical ventilation parameters: The new definition does not require a minimum PEEP level, increasing heterogeneity in ventilator management.

Limited validation for prognosis stratification in ventilated patients: The Berlin definition’s moderate-to-severe ARDS classification (PaO2/FiO2≤200 mmHg) is well-established, with multiple studies confirming its strong correlation with mortality (40-46% in moderate-to-severe ARDS patients). While the new definition retains PaO2/FiO2≤200 mmHg as the threshold for moderate ARDS, it expands the mild ARDS category to include PaO2/FiO2≤300 mmHg (S/F ≤ 315). However, this broader classification has not yet been validated in mechanically ventilated patients, raising concerns about dilution of effect size in high-risk subgroups.

3. Feasibility and Compatibility with Retrospective Database Studies

Timeframe mismatch: Our study is based on MIMIC-IV data (up to 2021), whereas the new ARDS definition was published in 2023. Using the Berlin definition ensures alignment with the data collection timeframe, preventing potential misclassification biases.

4. Strengthening the Study’s Conclusions

Our results indicate that higher mortality associated with TPDP >12.5 cmH2O was primarily observed in moderate-to-severe ARDS patients (PaO2/FiO2≤200 mmHg) as defined by the Berlin criteria. The Berlin definition’s stratification aligns well with ventilator-induced lung injury (VILI) mechanisms, including high driving pressure and mechanical power. If the new definition were applied (e.g., S/F ≤315), it would likely introduce more mild ARDS patients (PaO2/FiO2 201-300 mmHg). This might weaken the observed association between TPDP and mortality.

5. Supporting Evidence from Recent Literature

The continued use of the Berlin definition in recent research further supports its validity in mechanically ventilated ARDS patients. For example, a 2024 study by Ye et al. (Balkan Med J, 2025) analyzing ARDS mortality using MIMIC-IV still employed the Berlin definition (Ye D, Jiang W, Gu D., 2025). This further underscores its relevance in MIMIC-IV database studies.

In conclusion, the Berlin definition remains the optimal choice for this study due to its standardized PEEP requirements, validated prognostic stratification, and compatibility with retrospective data analysis. We have incorporated these justifications into the revised manuscript on page 12-13, line 391-403. Thank you again for your valuable feedback. We hope this revision adequately addresses your concern. Please let us know if further modifications or clarifications are needed.

Revised Text in the Discussion Section:

***The Berlin Criteria vs. the New Global Definition of ARDS***

*We selected the Berlin criteria due to its established integration with mechanical ventilation parameters. The 2024 Global ARDS Definition includes non-intubated patients and lacks standardized ventilation settings [30], whereas the Berlin criteria require PaO2/FiO2 assessment under PEEP ≥5 cmH2O, ensuring consistency. Additionally, moderate-to-severe ARDS classification (PaO2/FiO2 ≤200 mmHg) is widely validated for prognostic assessment, while the broader mild ARDS category in the new definition remains unverified in ventilated patients, increasing heterogeneity and potentially diluting effect size. Our findings reinforce this choice, as TPDP >12.5 cmH2O correlated most strongly with mortality in moderate-to-severe ARDS, a link consistent with ventilator-induced lung injury mechanisms. A recent MIMIC-IV study by Ye et al [31] continue to rely on the Berlin criteria, underscoring its relevance in mechanically ventilated patients.*

5. **Reviewer’ s comment: Statistical analysis--Regarding the statistical analysis of the work I go into the structural aspect of the work. All development points should be described under the big chapter of statistical analysis, possibly the other points will result subchapters of the same. To be properly restructured this section. The main section is the statistical analysis of the data.**

**Response:** I am grateful for this valuable feedback. In response to your suggestion, I have reorganized the statistical analysis section. All key points have now been grouped under a main chapter titled "Statistical Analysis." Subsections have been created to address specific aspects, such as Descriptive Statistics, Survival Analysis, Propensity Score Matching, Causal mediation analysis, and Cluster analysis. This restructuring ensures that the methodology is presented in a more logical and coherent manner, making it easier for readers to follow the different components of the analysis.

Revised Text in the Statistical Analysis Section:

***Statistical analysis***

*Data analysis was conducted using R version 4.3.1. The statistical methods were structured into the following subsections:*

***Descriptive Statistics***

*Continuous variables following a normal distribution are presented as mean ± standard deviation (MD±SD), and comparisons between two groups were conducted using the t-test. Continuous variables not following a normal distribution are presented as median (interquartile range) [Median(IQR)], and comparisons between two groups were conducted using the Wilcoxon test. Categorical variables are presented as percentages.*

***Survival Analysis***

*Survival curves were plotted using the Kaplan-Meier method, and differences in survival status between groups were compared using the Logrank test. The impact of transpulmonary pressure on the prognosis of ARDS patients was explored using univariate Logistic regression and univariate Cox proportional hazards regression models. A P-value < 0.05 was considered statistically significant. The optimal cutoff values for continuous variables in survival analysis were determined using the surv_cutpoint() function from the survminer package, based on Maximally selected rank statistics calculated by the maxstat.test function.*

***Propensity Score Matching***

*Propensity score matching (PSM) involves matching study subjects with similar propensity scores (PS) across groups to adjust for covariates, reduce confounding bias, and improve inter-group balance. Since the data used in this experiment were obtained from observational studies, confounding factors present among groups could bias the results. To minimize bias due to confounding factors, we used the MatchIt package in R4.3.1, employing the nearest neighbor matching method with a 1:1 match and a 0.05 caliper value for propensity score matching. Covariates included age, BMI, APSIII, SOFA score, diastolic and systolic arterial blood pressure (ABPd, ABPs), heart rate, WBC, blood glucose, creatinine, blood urea nitrogen, lactate, platelet count, prothrombin time (PT), arterial blood pH, PaO_2_, PaCO_2_, HCO3-, first-day oxygenation index, tidal volume, total respiratory rate, spontaneous respiratory rate, set respiratory rate, PEEP, plateau pressure, peak airway pressure, lung compliance, driving pressure, and mechanical power. After matching, there were 263 patients in the transpulmonary pressure target group and 263 patients in the non-target group. The balance between the two matched groups was assessed using the Standardized Mean Difference (SMD).*

***Causal mediation analysis***

*Causal mediation analysis (CMA) is a method that can divide the total effect of treatment into direct and indirect effects. It aims to identify and quantify the mechanisms between independent and dependent variables. If variable X affects variable Y through variable M, then variable M is called the mediator variable. After controlling for variable M, the effect of X on Y is the average direct effect (ADE). The effect of X on M and the effect of M on Y are collectively called the average causal mediation effect (ACME). In our study, we hypothesized that a specific indicator was a mediator variable. Transpulmonary driving pressure might lead to changes in this indicator, which are associated with mortality in patients with ARDS. The mediation effect was further explored by comparing PaCO_2_, HCO3-, plateau pressure and APSIII, PaCO_2_, peak airway pressure, plateau pressure, lung compliance, and driving pressure because of their differences between high and low TPDP groups and high and low transpulmonary driving pressure groups. ACME, ADE, and the total effect obtained by CMA were used to confirm our hypothesis that TPDP or transpulmonary driving pressure affects mortality through specific mediators.*

***Cluster analysis***

*Lasso regression was utilized to identify variables influencing ICU mortality in patients using transpulmonary pressure. Variables exhibiting right-skewed distributions were logarithmically transformed to stabilize variance and subsequently standardized. The optimal number of clusters and the distribution of K-means clustering under resampling conditions were assessed using consensus clustering (CC), with a subsampling ratio of 80%, 1000 iterations, and exploration of cluster numbers (k) ranging from 2 to 6. The best cluster number was determined by analyzing the heatmap of the Consistency Matrix (CM), the Cumulative Distribution Function (CDF), and intra-cluster consistency scores. An intra-cluster consistency score close to 1 indicated higher cluster stability. Once the optimal cluster was identified, the results were visualized using t-distributed stochastic neighbor embedding (t-SNE) and line graphs. We examined the association between clinical features of different clustering phenotypes and mortality, representing clinical features as percentages, mean±standard deviation, or median and interquartile range. Group comparisons were performed using the chi-square test, analysis of variance, and Wilcoxon’s test. All analyses were conducted using R version 4.3.1.*

The updated statement can be found on page 4-6, line 153-219.

6. **Reviewer’ s comment:**

**Discussion**

(1) **Line 331-381 This part of the discussion is a repetition, in some places, almost without difference, of the background described above. The discussion should “discuss” as the word says, the results obtained, all comparing them with the present literature and evaluating, point by point, whether in favor or different, looking for reasons, valid, supported by as much literature. Instead, in this part, the authors just rewrote something that was unnecessary. This part is all to be removed.**

**Response:** I truly appreciate this insightful feedback. In response, I have completely revised this section to focus on discussing the results obtained from our study. The redundant content has been removed, and I have restructured the discussion to compare our findings with the existing literature, carefully evaluating whether our results are in agreement with or differ from previous studies. Each result is now addressed point by point, with a critical review of the literature to support or contrast with our findings.

(2) **I advise the authors to restructure this part better, to really compare themselves with the literature, maybe even with some recent literature trying to properly evaluate their results. Perhaps an idea would be to take each result point by point and develop a discussion on them, point by point, with the appropriate literature to accompany the results and the discussion.**

**Response:** I have followed this suggestion and restructured the discussion to provide a more comprehensive comparison with the current literature, particularly focusing on recent studies. I have included a more detailed analysis of how our results compare with previous research, highlighting both similarities and differences, and providing possible explanations for these discrepancies, supported by the relevant literature.

Revised Text in the Discussion Section:

*Our study demonstrates that elevated transpulmonary driving pressure shows significant association with increased mortality in acute respiratory distress syndrome patients. Recent investigations have further underscored the critical prognostic implications the TPDP in ARDS management. Placenti et al. [22] established that TPDP more reliably reflects lung distension pressures compared to airway driving pressure, representing the actual transpulmonary pressure gradient without the distending pressure generated by the chest wall or esophageal driving pressure. In a study by Baedorf Kassis et al [23], treatment strategies leading to decreased transpulmonary driving pressure may be associated with improved 28 day mortality. This finding is consistent with the 2024 study by Sun et al, where TPDP should be used as a key target indicator for lung protective ventilation [24].*

*Our study further revealed that when TPDP exceeds 12.5 cmH₂O, patient mortality significantly increases. This threshold closely aligns with the 'safety window' (10–12 cmH₂O) proposed by Placenti et al. [22], suggesting that 12.5 cmH₂O may represent the critical threshold for triggering ventilator-induced lung injury (VILI). This relationship may be partially mediated through higher peak airway pressure. The detrimental clinical impacts of elevated peak airway pressures have been well documented. Experimental evidence from a rat mechanical ventilation model developed by Katira et al. [25] revealed that excessive peak airway pressure induce right ventricular overload through increased pulmonary vascular capacitance and perfusion abnormalities. It is suggested that control of peak airway pressure can interrupt the vicious cycle of "high TPDP→ alveolar hyperinflation → pulmonary circulation injury". Our causal mediation analysis substantiates the hypothesis that therapeutic strategies targeting TPDP reduction could mitigate both peak airway pressure elevation and subsequent pulmonary damage. This mechanistic understanding emphasizes the potential clinical utility of TPDP-guided ventilation strategies in ARDS management.*

*The differential effects of transpulmonary driving pressure (TPDP) across ARDS phenotypes arise from distinct pathophysiological mechanisms and heterogeneous lung mechanical properties. In Phenotype I (High Mechanical Power with Moderate Lung Compliance), the preserved lung compliance indicates partial alveolar recruitability. However, excessive mechanical power potentiates VILI via bioenergetic stress-induced tissue damage and inflammatory cascades[26], where elevated TPDP potentiates ventilator-induced lung injury through biomechanical transduction pathways. Phenotype II (High Spontaneous Effort with Elevated Compliance) demonstrates paradoxically increased lung compliance combined with vigorous spontaneous breathing effort, creating conditions favorable for pendelluft phenomenon[27]. In this context, TPDP elevation amplifies cyclic alveolar collapse-reopening cycles in dependent lung zones, thereby exacerbating atelectrauma through shear stress mechanisms[28]. Phenotype III (Low Tidal Volume with Reduced Compliance), characterized by low tidal volume and markedly diminished compliance, exhibits limited TPDP transmission due to mechanical stress dissipation through non-recruitable lung parenchyma. This stress-shielding effect explains the absence of significant mortality correlation despite TPDP fluctuations. This mechanistic stratification underscores the clinical imperative for phenotype-driven ventilation strategies. Our observations harmonize with contemporary evidence from Liu et al. [29], reinforcing the paradigm shift toward precision medicine in ARDS management.*

The updated statement can be found on page 11-12, line 342-390.

7. **Reviewer’ s comment: Conclusions--I would advise the authors to be softer and softer with the conclusions, it is important, even considering the limitations of the study, to try to use the conditional and carefully evaluate the words in the conclusions of the study. To be corrected.**

**Response:** I have carefully revised the conclusions section to use more cautious language. I have incorporated conditional statements and emphasized the limitations of the study to ensure a more balanced and measured conclusion. Revised Text in the Conclusions Section:

*Our study suggests that TPDP >12.5 cmH2O may be associated with an increased risk of mortality in ARDS patients, particularly those with moderate to severe ARDS. Incorporating target TPDP values into routine clinical practice might enhance the precision of mechanical ventilation strategies, potentially reducing ventilator-induced lung injury (VILI) and improving patient outcomes. However, recognizing the different ARDS phenotypes and tailoring ventilation strategies based on these profiles could further contribute to better patient prognosis. Future research should aim to validate these findings, develop phenotype-specific management protocols, and explore the mechanisms underlying the varied responses to TPDP in ARDS patients. This will help provide a more comprehensive understanding of the role of TPDP in the management of ARDS.*

The updated statement can be found on page 13-14, line 435-445.

8. **Reviewer’ s comment: Figure 1--I advise the authors to avoid color and focus mainly on the accuracy of the concepts they want to get across, the first BOX is not clear, which patients were identified by MIMIC? In the BOX where the authors state the patients meeting the Berlin criteria for ARDS is the number to be entered first, since the others were all structured this way, as were the other two groups, either all with n in parentheses, or all with numbers at the beginning. To be restructured properly and logically in the figure.**

**Response:** Thank you for your valuable suggestion. In response, we have made the following revisions to Figure 1: We have redesigned the flowchart, where the first box represents the total number of patients included in the MIMIC-IV database. The second to the fourth boxes show the patient selection process according to the inclusion criteria. We have removed the use of color and focused on improving the accuracy and clarity of the concepts. And we have also put all numbers at the beginning. Here is the revised flowchart.

9. **Reviewer’ s comment: Figure 2-- I don't find this figure useful, in fact, it seems to me almost a given that if the patient has a P/F <100 they are more likely to die, or less likely to survive, depending on how we want to look at it, than a patient with a PF between 200 and 300, what is the reason for including this figure?**

**Response:** Thank you for the reviewer’s insightful comment. You have a sharp perspective—indeed, patients with PaO2/FiO2 <100 mmHg have a higher mortality rate. However, our intention with Figure 2 was to highlight the differences in mortality across ARDS severity groups and set the stage for Table 2 and S1 Table, which further explore how transpulmonary driving pressure (TPDP) varies across different oxygenation levels. This figure establishes the foundational stratification, helping to contextualize the subsequent analysis on TPDP-related differences among ARDS patients. That said, in response to your comment, we have relocated Figure 2 to the supplementary materials to ensure better alignment with the manuscript’s key focus while maintaining transparency in data presentation. We appreciate your thoughtful suggestion and believe this adjustment improves the manuscript’s clarity and coherence. Thank you again for your valuable feedback.

10. **Reviewer’ s comment: Figure 3--Interesting KM at 90 days on survival, it would also be interesting to have one, equal to 28 or 30 days, to better understand the difference, maybe trying to “zoom in” excuse the term, removing the unnecessary part of the graph. I don't know if I explained myself. In the end, it turned out a figure that the authors took into consideration, the 28-day mortality/survival if I remember correctly.**

**Response:** Thank you for the reviewer’s insightful comment regarding the Kaplan-Meier (KM) survival curve at 90 days. We appreciate the suggestion to include a 28-day survival analysis to further explore potential differences. To clarify, our study primarily focused on ICU mortality as the primary outcome. However, in response to the reviewer’s suggestion, we conducted an additional analysis of 28-day mortality. The results showed no significant difference in survival at 28 days. Given this finding, we included the statistically significant ICU mortality KM curve in the main text, while the 28-day KM curve has been moved to the supplementary materials (Table S2) for reference. Additionally, we performed component comparisons for 28-day mortality, and both group comparisons and Cox regression analysis did not show significant differences. As a result, we have placed these results in the supplementary materials rather than the main text. We appreciate the reviewer’s suggestion, as it helped us further validate our findings. The relevant updates can be found in S3 Table of the supplementary materials. Please let us know if further clarification is needed.

Figure 3 28-day survival analysis of TPDP group and No-TPDP group after matching.

We hope our responses and revisions address all concerns raised by the reviewers. Please feel free to contact us if there are additional questions or suggestions. Thank you for your time and consideration.

Sincerely,

Mingxing Fang

m18533112886@hebmu.edu.cn
