# Supplementary material for: The effect of target transpulmonary driving pressure values on mortality in ARDS patients: A retrospective study based on the MIMIC-IV database
Source: PLoS One. 2025 Jun 18;20(6):e0326060. doi: 10.1371/journal.pone.0326060 (PMC12176163; doi:10.1371/journal.pone.0326060)
Supplement: Attachment — Submitted filename: PLOSONEPoint-by-point_response_auresp_3.docx [file pone.0326060.s019.docx]

Dear Editors and Reviewers:

Thank you for your letter and for the Reviewers’ comments concerning our manuscript entitled “The Effect of Target Transpulmonary Driving Pressure Values on Mortality in ARDS Patients: A Retrospective Study Based on the MIMIC-IV Database”. (Submission ID PONE-D-24-38166R1). Those comments are all valuable and very helpful for revising and improving our paper, as well as the important guiding significance to our researches. We have studied comments carefully and have made correction which we hope meet with approval. Revised portion are marked in red in the paper. The main corrections in the paper and the responds to the reviewer's comments are as following:

**Responses to the Reviewer 1’s comments:**

1. **The authors have done an excellent job of addressing my questions and revising the manuscript. I would like to emphasize that evidence supporting the hypothesis about the effects of transpulmonary driving pressure in this manuscript are indirect. Proof of concept will require animal model experiments. The authors are obligated to mention this fact to the readers.**

**Response:** We sincerely appreciate your thoughtful and supportive comments on our revised manuscript. We are grateful for your recognition of our efforts in addressing your previous concerns. We acknowledge and fully agree with your important point that the current evidence supporting the role of transpulmonary driving pressure (TPDP) in ARDS is indirect and observational. As you rightly noted, proof-of-concept studies using animal models or prospective interventional trials are needed to establish a causal relationship. Accordingly, we have revised the Discussion section of the manuscript to explicitly state this limitation and emphasize the need for future mechanistic studies. Specifically, we have added the following sentence:

*“It should be noted that our findings are derived from retrospective observational data, and the evidence supporting the role of transpulmonary driving pressure in influencing mortality is indirect. Causality cannot be inferred from these results. Therefore, proof of concept studies using animal models and prospective interventional trials are needed to validate the physiological mechanisms and causal links suggested by our findings.”* The updated sentence can be found on page 22 line 419-425.

We hope this revision adequately addresses your concern and improves the clarity and scientific rigor of our manuscript.

**Responses to the Reviewer 2’s comments:**

1. **The authors have made significant improvements in addressing the concerns raised in the previous review. The manuscript is now technically sound, clearly written, and well-organized. That said, I would like to emphasize that the conclusions drawn regarding transpulmonary driving pressure as a contributor to mortality are based on indirect evidence from observational data. While the findings support the hypothesis that elevated transpulmonary driving pressure is associated with higher mortality in ARDS, this does not constitute proof of causation. Proof of concept would require further validation, ideally through animal model experiments or prospective interventional trials. I recommend the authors explicitly state this limitation in the Discussion or Conclusion section to ensure readers are aware of the inferential nature of these results. Therefore, I support publication pending minor revision to clarify this key point.**

**Response:** Thank you very much for your thoughtful and encouraging comments. We sincerely appreciate your recognition of the improvements made in the manuscript and your support for its publication. We fully agree with your observation that the association between elevated transpulmonary driving pressure (TPDP) and mortality in ARDS, as demonstrated in our study, is based on retrospective observational data and thus does not establish a causal relationship. As you rightly pointed out, proof of concept would require validation through animal models or prospective interventional trials. In response to your suggestion, we have revised the Discussion section of the manuscript to explicitly acknowledge this limitation. The following sentence has been added:

*“It should be noted that our findings are derived from retrospective observational data, and the evidence supporting the role of transpulmonary driving pressure in influencing mortality is indirect. Causality cannot be inferred from these results. Therefore, proof of concept studies using animal models and prospective interventional trials are needed to validate the physiological mechanisms and causal links suggested by our findings.”* The updated sentence can be found on page 22, line 419-425.

We hope our responses and revisions address all concerns raised by the reviewers. Please feel free to contact us if there are additional questions or suggestions. Thank you for your time and consideration.

Sincerely,

Mingxing Fang

m18533112886@hebmu.edu.cn
